# A record of seafloor methane seepage across the last 150 million years

**DOI:** 10.1038/s41598-020-59431-3

**Published:** 2020-02-13

**Authors:** D. Oppo, L. De Siena, D. B. Kemp

**Affiliations:** 1 School of Geosciences, University of Louisiana at Lafayette, Lafayette, LA 70503 USA; 2 Institute of Geosciences, Johannes Gutenberg University Mainz, D−55128 Mainz, Germany; 3 School of Earth Sciences and State Key Laboratory of Biogeology and Environmental Geology, China University of Geosciences, Wuhan, 430074 China

**Keywords:** Biogeochemistry, Ocean sciences, Ocean sciences

## Abstract

Seafloor methane seepage is a significant source of carbon in the marine environment. The processes and temporal patterns of seafloor methane seepage over multi-million-year time scales are still poorly understood. The microbial oxidation of methane can store carbon in sediments through precipitation of carbonate minerals, thus providing a record of past methane emission. In this study, we compiled data on methane-derived carbonates to build a proxy time series of methane emission over the last 150 My and statistically compared it with the main hypothesised geological controllers of methane emission. We quantitatively demonstrate that variations in sea level and organic carbon burial are the dominant controls on methane leakage since the Early Cretaceous. Sea level controls methane seepage variations by imposing smooth trends on timescales in the order of tens of My. Organic carbon burial is affected by the same cyclicities, and instantaneously controls methane release because of the geologically rapid generation of biogenic methane. Both the identified fundamental (26–27 My) and higher (12 My) cyclicities relate to global phenomena. Temporal correlation analysis supports the evidence that modern expansion of hypoxic areas and its effect on organic carbon burial may lead to higher seawater methane concentrations over the coming centuries.

## Introduction

Methane is a powerful greenhouse gas emitted into the atmosphere by numerous natural and anthropogenic sources. Natural methane leakage from the seafloor (i.e. cold seeps) is a well-known phenomenon occurring in a wide range of geologic and geodynamic settings, including deep-sea fans, convergent margins, and polar regions, with new seepage sites steadily discovered across all oceans^1–3^. Marine cold seepage systems are complex and highly dynamic, and can respond rapidly to external perturbations such as variations in pressure and temperature^4^. This may lead to the release of large methane volumes and have a relevant impact on both local environment and the global climate, as has been suggested to explain extreme events in the geological past^5^. Among the various natural methane origins, marine sources play a first order role because sub-seafloor sediments host enormous volumes of this gas^6,7^. Sources of seafloor methane include maturation of organic matter, gas hydrates dissociation, sill-induced release of sedimentary carbon, or volcanic activity^4,8,9^. The total modern emission of seafloor methane is likely underestimated^10^ and the volumes of methane released at the seafloor are orders of magnitude higher than those reaching the sea surface, owing to the short residence time of methane in seawater^11,12^. The volume of methane released from the seafloor is reduced also via microbial Anaerobic Oxidation of Methane (AOM)^13^, which consumes an estimated 45–61 Tg∙y^−1^ in the shallow sub-seafloor^6,14^. The AOM process is of primary importance since it provides a significant mechanism to decrease the volume of escaping methane^10^ and leads to the precipitation of methane-derived carbonates (MDC) as a by-product^15^, thus representing a carbon sink in the sedimentary record^16,17^. As a result, understanding past and present dynamics of methane release and MDC formation in ocean sediments is valuable for advancing climate change predictions^8^. Defining the processes involved in long-term cold seepage is also necessary for establishing the precise relationship between geological (e.g., tectono-stratigraphic history and climate oscillations) and biochemical processes (e.g., generation of hydrocarbon and their degradation)^18^.

**Figure 1:**
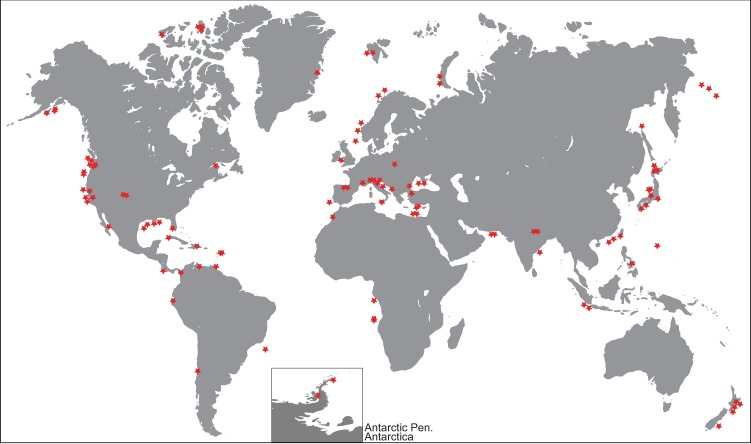
Location of the methane-derived carbonates used in this study. World map showing the occurrences of methane-derived carbonates used to compile the time series in this study. Only one representative sample for each location/age combination has been considered (Methods M1a). Map generated with ESRI ArcMap (version 10.6.1, https://desktop.arcgis.com/en/arcmap/).

**Figure 2:**
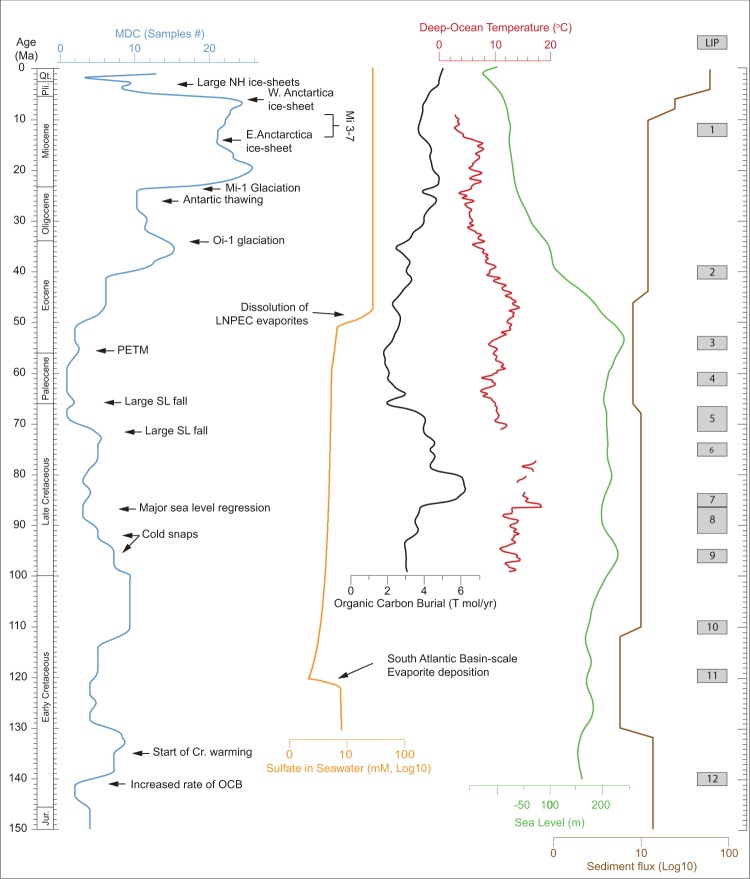
Time series of methane-derived carbonates and possible controllers during the last 150 My. Records of modelled Organic Carbon Burial (OCB)^25^, global sea level^23^, deep-sea water temperature^24^, seawater sulfate^59^, global sediment flux^60^, and large igneous provinces (LIP)^23^ are also shown for comparison. The MDC, OCB, sea level, and the temperature time series are interpolated to a 1 My time interval for the purposes of statistical analysis. The main climatic events are shown. Large Igneous Provinces (LIPs^23^): 1 Iceland; 2 Kerguelen (N), Walvis (S); 3 Mascarene; 4 Dal Cano; 5 Crozet, Walvis (C); 6 Magellan, Maud; 7 Kerguelen (Elan); 8 Broken R, Caribbea, Rio Grande, Conrad, Madagascar, Walvis (N); 9 Wallaby; 10 Hess, Kerguelen(S); 11 Manihiki, Hikurangi, Ontong Java; 12 Shatsky.

Studies on seafloor methane seepage have primarily focused on either ancient or modern examples without full integration of the two in a long-term seepage history model. However, we know that cold seepage systems can be regionally active on multi-million year time scales in dynamic geological and climatic settings^19^. An exhaustive knowledge of the processes that may control methane release on long, geological time scales is still missing. Rates and temporal patterns of seafloor methane emission have varied through geological time, but few studies have attempted to quantify the long-term emission history using large-scale compilations of globally distributed data^20^. In this study, we have compiled a database of worldwide occurrences of MDC for which accurate dating is available (Fig. 1). We use these data to reconstruct the history of global MDC occurrence and natural methane emission from the seafloor across the last 150 My (Fig. 2). We then selected data available in the literature to test the relative importance of global changes in sea level^21–23^, deep ocean temperature^24^, and organic carbon burial^25^ in mediating long-term methane release. We performed this test first on the past 100 My due to length limitations of the other curves considered. Further comparisons and analyses were performed for longer periods if longer time series were available.

## Multi-Million-Year Proxies for Methane Seepage

Estimating seafloor methane emission is challenging because of the poor preservation of past seepage evidence in the sedimentary record^26^ and because thousands of highly dynamic seepage sites are projected to be discovered over the coming decades^11^. Various geophysical, geochemical, and mineralogical proxies have been used to reconstruct the history of methane release from the seafloor. Geophysical surveys (e.g. reflection seismic) allow recognition of pockmarks and mud volcanoes on the modern seafloor and in the subsurface, which have been associated with discrete events of past methane release^27,28^. However, their occurrence in the fossil record is rare in the literature^29–31^, demonstrating a low preservation potential. The formation of gas hydrates has been investigated in various regions of the world^32^ and is often recognised in seismic data though a bottom-simulating reflector (BSR) that marks the contact between possible gas hydrates (above) and free gas (below). When two separate BSRs at different depths within the subsurface are found, they can indicate a shift of the gas hydrates stability zone (GHSZ), implying hydrate dissociation and re-formation in a different portion of the sediment column^33^. The use of BSR to reconstruct methane seepage is limited and is weakened by its possible absence, even in the presence of hydrates^34^. Diagnostic minerals relate to variations in the GHSZ^32^, i.e. barite fronts indicating the top of the methane-bearing zone, can be preserved during periods of increased methane hydrate stability and thus reduced methane flux^35^.

Geochemical analyses have mainly focused on the δ^13^C signature that methane transfers to the mineral phases, with negative excursions of δ^13^C observed in sediments of both marine and terrestrial origin that have been often used as a proxy of important methane release^36,37^. Anaerobic^38^ or aerobic^39^ methane oxidation involves various methanotrophic microbial consortia. Lipid biomarkers and their stable isotopes have been used to reconstruct the past occurrence of microbial methane oxidation in fossil cold seeps thanks to the significant preservation of the biomarker inventory^17,40^. The dynamics of microbial activity is related to the variation of methane source and flux, which is in turn reflected by the lipid biomarker record^41,42^. Despite lipid biomarkers having the potential to record variations of methane through time, they do not provide information on the absolute age when these variations occur and thus their best application is to reconstruct dynamics at local scales. The use of δ^13^C can also be applied to overgrowths of authigenic carbonate on benthic foraminifera tests^43,44^. This method can potentially provide high-resolution records of methane seepage, but its suitability is a subject of current debate^43^. In particular, there is still a need to clarify the respective contributions to δ^13^C from primary foraminiferal calcite, reflecting the environmental dissolved inorganic carbon during the living stage, and secondary authigenic calcite overgrowths formed on dead tests^45^. In addition to lipid biomarker and stable isotope records, the concentration of heavy hydrocarbons (i.e. tar) has been used as a proxy to estimate methane seepage and changing concentration of petroleum compounds, such as within Late Quaternary sediments in the Santa Barbara Channel^46,47^.

The use of the above-mentioned methods as proxies for methane emission on long geological time scales is linked to clear uncertainties and limitations. A potentially more useful and robust proxy is that of MDC. Between 10% and 20% of methane oxidised by AOM precipitates as carbonate minerals^15^, which are the main by-products of this process in marine environments^17^. Because MDC are documented in sedimentary units ranging from the Neoproterozoic^48^ to Present^49^, both modern and fossil MDC potentially record the trend of seafloor methane seepage across large intervals of geological history. MDC can yield an absolute age for their formation either by dating of the authigenic cements themselves^50,51^ or, indirectly, by the age of the host sediments^16^.

The use of MDC to compile a record of seafloor methane seepage spanning tens of million years presents some challenges, mainly associated with the possibility of bias in sample preservation and accessibility. MDC formation within sub-seafloor sediment can limit their ability to be identified if still buried below the modern seafloor. The MDC record can also be influenced by the preservation potential of rock related to age^52^, with a possible decrease in sample number further back with time. Bias associated with rock volume preservation has been investigated mainly in paleobiodiversity studies, and the use of sampling proxies to correct the data has been applied^53^. The use of proxies such as outcrop area and gap-bound sequences^54,55^ cannot be readily applied to our worldwide compilation of MDC because of the great variability of the dataset itself. Otherwise, the formation count proxy^56^ can be applied by assuming that all MDC-bearing formations contain an equal number of samples, a simplification that has been used in similar studies^20^. One additional bias in the use of MDC could be the concentration of sulfate in seawater because its involvement in the carbonate precipitation process (see the next sections for a detailed discussion of the role of sulfate). It is also expected that new MDC will be discovered in the future, in particular in the increasingly explored polar regions. Nevertheless, compiling an extensive dataset of worldwide MDC occurrences within sediments and rocks spanning 150 My mediates at a global scale the effect of local and regional factors that may affect methane release, such as tectonic activity or ice sheet dynamics.

## Statistical Analysis, Validation, and Analytical Reconstruction of the MDC Record

**Figure 3:**
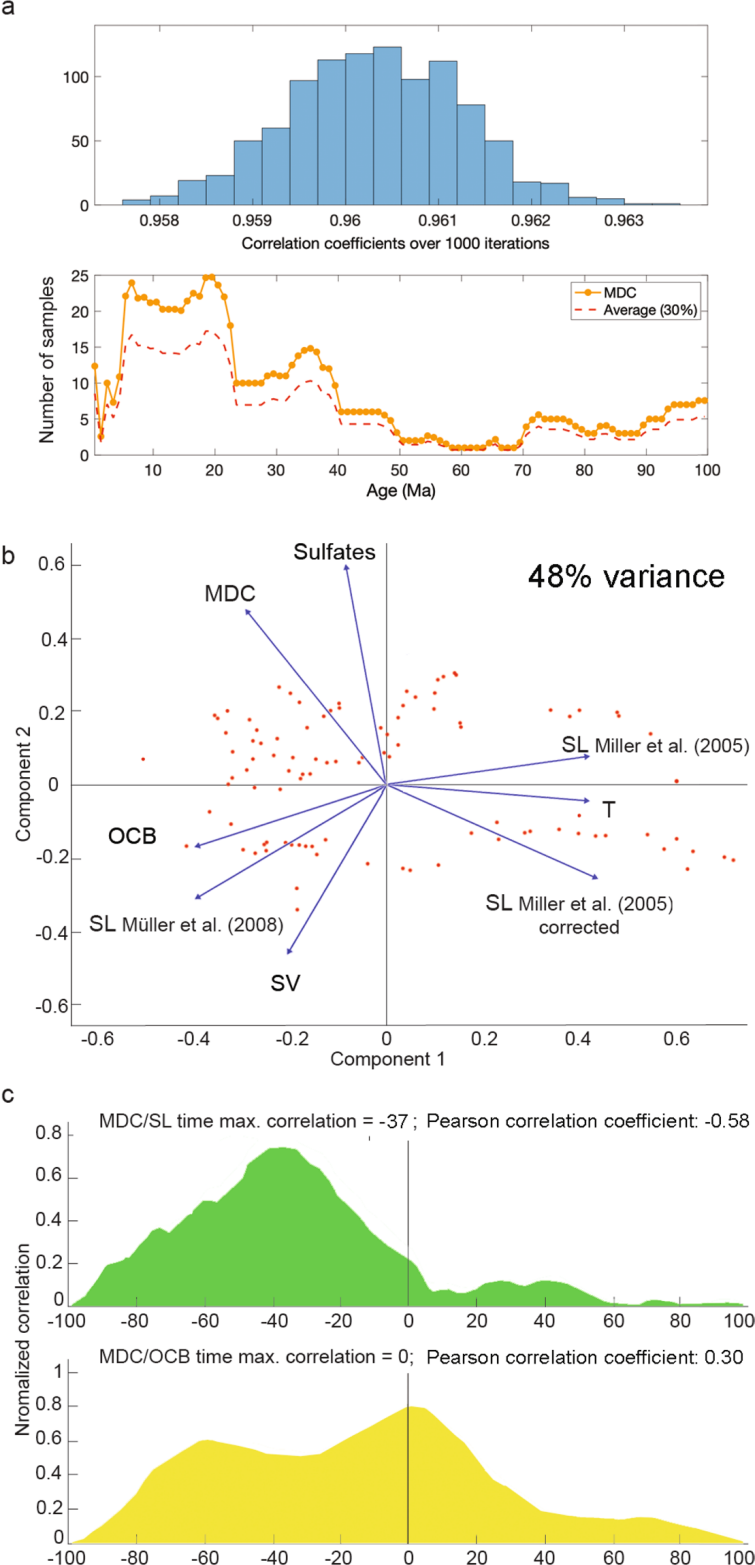
Statistical analyses of methane derived carbonate abundance (MDC) and associated time series. (**a**) Results of a 30% bootstrap test of the MDC time series. The histogram calculated over 1000 resampling (upper panel) shows the normalised correlation coefficients with respect to the original curve. The red dashed line (lower panel) is the average of these curves, showing how both the gross features and the peaks of the time series curve are preserved. (**b**) Principal component analysis performed on the ensemble of available measurements and models. Scores are shown as red dots. SV: sediment volume, SL: sea level, T: temperature. See Figs. S1-S2 for comparisons of different components and datasets. (**c**) Time-dependent normalised cross-correlation between MDC and sea level and OCB residuals. The value and time-lag of the maximum of the cross-correlation is highlighted at the top of each panel. The peak at negative times in all cross-correlation is due to the finite length of the considered time series.

The MDC record presented in this work is a robust time series built from a global database with sampling time 1 My (Methods, M1-a) (Fig. 3a). The sampling time must be larger than the time methane requires to generate MDC and depends on the uncertainties in the dating of the samples and their distribution in time. The time necessary to generate an MDC is in the order of tens of thousands of years for massive concretions^57^, thus well within the chosen sampling step (i.e. resolution of our MDC abundance curve). The decreasing number of MDC samples retrieved going back in time suggests that the only trend required to model MDC data is a linear decrease (Methods, M1-b). The residuals of the MDC record with respect to this trend are non-stationary, i.e. they have no long-term mean and are variable over the last 150 My (Methods, M1-b). This means that standard correlation analysis procedures (e.g., Pearson coefficients) offer only partial information when comparing MDC occurrence with likely controllers, even after de-trending. Stochastic shocks or cyclicities make the process not mean-reverting^58^. This further implies that it is challenging to interpret single peaks or troughs of the MDC record and correlate them with coeval variations in its likely controller, as they could be due to underlying periodic signals and measurement errors.

**Figure 4:**
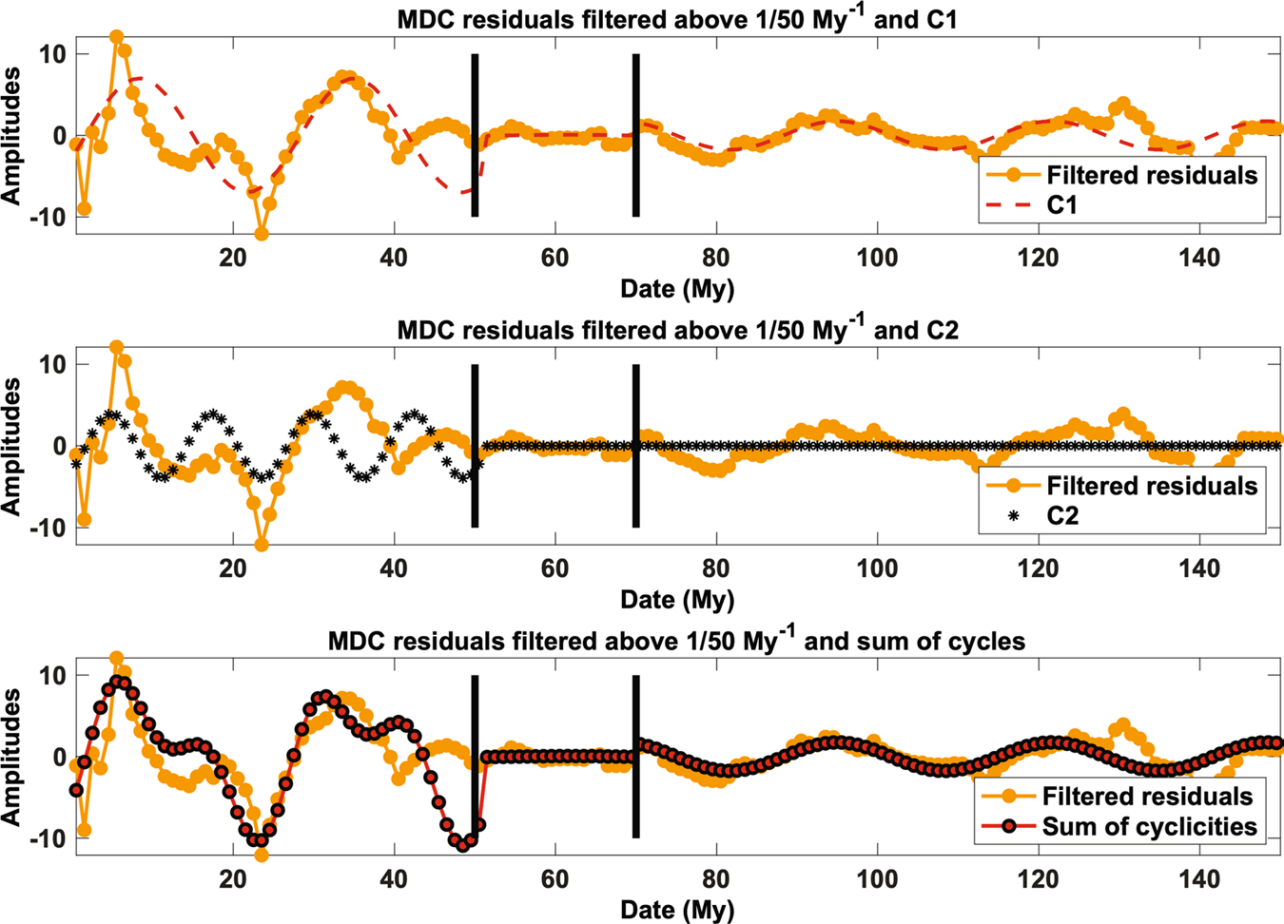
Autocorrelation and residuals analysis. Reconstruction of the entire residual fit of the MDC record from its two recognised cyclicities after high pass filtering (cut-off frequency: 1/50 My^−1^). The divergences at ~50 Ma and ~130 Ma are associated with major excursions in sulfate concentration (see Fig. 2).

We can broadly characterize Earth-related signals (e.g. MDC) with spectral analyses as those used in standard time series and signal processing, in order to understand if and when periodic signals (from now named “global cyclicities”) affect the records. The spectrograms (Methods 1-b - Supplementary Figs. S4–S6) reveal a first cyclicity (C1 = 1/26.66 My^−1^) that is stable across the last 150 My as well as an out-of-phase cyclicity (C2 = 1/12 My^−1^) necessary to reconstruct the MDC linear residuals after 70 My(Fig. 4). This analytical model is a first-order predictor of methane seepage emission before the Anthropocene: in this model, C1 characterises the entire MDC record, while C2 (amplitude > 1/2 C1) activates after 70 Ma and lasts until the Present (Supplementary Fig. S6). Apart for the loss of apparent cyclicity in the interval 50–70 My, the analytical signal follows an opposite behaviour compared to the detrended MDC curve between 40 and 50 Ma and 130–140 My (Fig. 4, lowermost panel).

## The MDC Record and its Likely Controllers

To statistically test the likely controllers on our MDC time series, we employed principal component analysis (PCA) of the MDC data along with data compiled from the literature on 1) global seawater sulfate concentration^59^, 2) global sea level^21–23^, 3) deep sea global temperature^24^, 4) global sediment flux^60^, and 5) organic carbon burial^25^ (Fig. 2). PCA was performed on linearly detrended data (see Methods, M2, Fig. 3b and S2). PCA results demonstrate that only variations in sulfate, global sea level, and organic carbon burial (OCB) contribute consistently and significantly (p < 0.02) to MDC variance.

Sulfate availability in seawater has been linked to the formation of hydrate-related MDC because sulfate reduction is required for AOM and thus MDC formation^59^. This causal relationship is shown in the PCA, where it can be demonstrated that sulfate has the strongest positive correlation with MDC (Fig. 3b). As with sea level and OCB, sulfate shows a long-period cyclicity of ~70 My wavelength (Methods M2). This single period is caused by the major sulfate changes at ~50 Ma and ~120 Ma (Fig. 2). Sulfates lack cyclicities below wavelengths of 50 My that characterize MDC, sea level, and OCB (Fig. 2) and thus do not provide any significant information to explain C1 and C2. These cyclicities can therefore be more plausibly linked to OCB and sea level variations. In Fig. 4, we show that we can reconstruct almost the entire residual fit of the MDC record by summing the C1 and C2 sinusoids, using the amplitude ratios and phases determined by the spectra (see Methods M1c). The only major discrepancies between the residual fit and the sum of cycles are at ~50 Ma and ~130 Ma, corresponding to the two major variations in the sulfate curve, which relate to major events of evaporites dissolution and precipitation, respectively^59^ (Fig. 2). These two discrepancies are therefore due to the two geological rapid changes in seawater sulfate concentration.

During sea level decrease, methane gas trapped in the subsurface is subject to lowered hydrostatic pressure owing to the reduced thickness of the overlying water column. Gas is thus more likely to migrate upwards through buoyancy. A decrease in pressure also favours a shift of the GHSZ, possibly destabilising methane hydrates and adding to the volumes of gas released^61^. Thus, the observed anti-correlation of MDC and sea level (Fig. 3b) is expected^20^. We tested this hypothesis using the sea level curves of Miller *et al*.^21,22^, Müller *et al*.^23^, and the Pure Backward Advection Curve of Müller *et al*. (Miller *et al*. 2011 corrected) (Supplementary Fig. S1). The anti-correlation of MDC and sea level is significant only for the corrected curve (the squared correlation coefficient *r*^2^ is 0.34, even if the non-stationarity of the curves makes this number unreliable). We present our results considering the Miller *et al*. corrected curve^23^ because it shows the best covariance of the curve variations in the principal component analysis.

A positive correlation between OCB and methane generation had been observed acting locally and on short temporal scales^10^. In contrast, the long-term (tens of My) correlation between modelled OCB^25^ and MDC observed in this work has not been clearly demonstrated before. As with the MDC data, both OCB and the sea level time series used are non-stationary over the last 100 My (Methods, M1-M2). Once common low-period signals (> 50 My) are filtered out (Supplementary Fig. S3), the spectral analyses show that sea level and OCB share the same cyclicities as the MDC record (Supplementary Fig. S8). Cyclicity C1 has a frequency of 1/26.66 My^1^ and is pervasive across the whole 140 My (sea level) and 100 My (OCB) time series. C1 is dominant at ages greater than 70 Ma in both the MDC and sea level records, which are the only two time series extending longer than 100 My. The cyclicity C2 = 1/12 My^−1^ occurs after 70 Ma and is shared by the MDC and OCB records.

It is necessary to assess if normalised correlations are time-lag dependent (i.e. can be improved by shifting the curves in time) to determine more precisely the temporal relationships between MDC occurrence and both sea level and OCB. This assessment can also help to clarify the geological significance of delayed responses between cause and effect (e.g. time delay required for methane generation following organic matter deposition). The temporal correlation analysis shows that the MDC/Sea level time correlation is most significant tens of My before or after the zero-lag correlation (Fig. 3c). This correlation rises with a maximum at 37 My. This time shift is too long to show a meaningful correlation between sea level and seep carbonate formation. The peak is likely due to the finiteness of our datasets (Supplementary Material; Methods, M2). The analysis proves that no short-time correlations can be found. Differently, the OCB time correlation reaches an absolute maximum at 0 My (with a peak uncertainty of about 2 My – Fig. 3c). The maximum correlation at 0 My suggests that any causal relationship between OCB and MDC is coeval (i.e. geologically instantaneous), while sea level mainly affects long-range, smooth trends in MDC.

## Qualitative Relations of MDC with Sea Level and OCB

The record of MDC in the Early Cretaceous interval shows two periods of inferred increased methane expulsion at ~135 Ma and ~105 Ma (Fig. 2). The increase of OCB hypothesized right after the Jurassic-Cretaceous boundary^62^, associated with low in sea level and the initial phases of the Cretaceous greenhouse warming, may be in part responsible for the oldest of the MDC abundance peaks (Fig. 2). A negative peak in carbon stable isotopes has been identified in the Aptian and associated with extensive dissociation of methane hydrates^63^. However, this interval is characterized by a decrease in the MDC time series. A reduction of AOM efficiency is expected with progressive increase of methane flow, from 80% to less than 10% in the case of rapid degassing, which overwhelms the microbial oxidation capacity^11^ and, therefore, lowers MDC precipitation.

Our data reveal an overall decrease in MDC abundance from the Early Cenomanian (100 Ma) to the Late Paleocene (58 Ma; Fig. 2). Marked warmth and relatively high sea level started in the Albian and culminated with the Cretaceous Thermal Maximum (~85–90 Ma). During this period, two cold events likely led to the formation of short-lived ice sheets and consequent rapid sea level fluctuations at 96 Ma and 93–92 Ma^22^. These “cold snaps” are possibly recorded in our dataset by two breaks in the decreasing trend of MDC. During the second half of the Late Cretaceous, the MDC record correlates with a general stability in sea level on multi-My time scale resolution. A rising trend of MDC abundance culminated at 72 Ma, likely because of the large sea level fall at the Campanian/Maastrichtian boundary^22^. This peak in MDC precedes a sudden drop leading to a 10 My-long (68–58 Ma) curve minimum. The MDC decrease that initiated at 72 Ma is marked by a relevant sudden drop (~2 T mol/yr) in modelled OCB. An increase between 65 and 64 Ma is associated with a peak in OCB. We suggest that, in this case, organic matter burial may have a dominant role in controlling the rapid variations of the flux of methane in the shallow subsurface and through the seafloor.

A progressive overall increase in methane emission between 65 Ma and 6 Ma characterises the Cenozoic (Fig. 2). A small and transient MDC increase occurs across a time interval encompassing the Paleocene-Eocene Thermal Maximum (PETM) (56–55 Ma). Various hypotheses have been formulated to explain the start of PETM and the rapid massive emission of fossil carbon^64^, involving dissociation of gas hydrates^65,66^, organic matter oxidation^67^, permafrost thawing^68^, and extensive volcanic activity^9,37,69^ which led to the overall predominant emission of CO_2_^70^ with respect to methane. The PETM was short-lived event, in the order of ~ 200 ky^37,69^, which may not be well recorded in our curve that has only million year resolution. Additionally, the lower AOM efficiency in the presence of high methane fluxes could have had a role in the smaller than expected MDC precipitation. Interestingly, the two main gas hydrate release events within the analysed time interval (Aptian and PETM) are associated with a low abundance of MDC, thus further supporting this interpretation.

The increase in MDC starting at ~55 Ma is associated with initial phases of cooling at high latitudes, a major sea level regression^71^, and an increase of sulfate in seawater^59^ (Fig. 2). Whilst the general increasing trend is maintained throughout the Cenozoic, three cycles of increasing-decreasing MDC abundance are observed between 41 Ma and 6 Ma. Methane volumes progressively increased in the Late Eocene, with a maximum at 35 Ma. The reasons for the transient, but marked, decrease of MDC occurrences between 35 and 23 Ma, in association with an overall increasing OCB and decreasing sea level, are unclear. Between 27 and 23 Ma, a decrease in OCB likely reduced the probability of MDC formation. At the Oligocene-Miocene boundary the renewed preservation of organic matter and the sea level drop associated with the Mi-1 glaciation triggered a second increase in MDC.

During the 20–14 Ma interval, MDCs progressively decrease along with OCB, and corresponds to a rise in sea level associated with the Mid-Miocene Climatic Optimum^72^; these conditions are favourable to a reduction in methane emission. The initial re-glaciation of Antarctica (14 Ma) and the Mi-3 to Mi-7 glaciations mark the start of a new phase of more intense methane emission between 13 and 6 Ma. The main Miocene sea level fall (~25 m drop) at 11 Ma (associated with the establishment of a permanent East Antarctic Ice Sheet^71^) likely contributes to sustain methane emission during this period. During the 9–6 Ma interval, the widespread increase in MDC was likely a consequence of general sea level lowering associated with the formation of the West Antarctic Ice Sheet and the initial growth of ice sheets in the northern hemisphere^72^. Since the Early Pliocene (5 Ma), MDC formation has apparently drastically reduced, and contrasts with both a rise in sea level and a decrease in OCB over the same interval. However, we note that MDCs younger than ~1 Ma generally lay in proximity of the modern seafloor, and those older than ~6 Ma are mainly recorded in outcrops, making both of these occurrences easier to access and quantify. In contrast, MDCs with an intermediate age are most likely buried below the modern seafloor and consequently are unlikely to be observed. We thus suggest that the trend of this portion of the curve is biased by the scarce accessibility of MDCs with ages between ~1 and ~6 Ma. Note that MDCs occurring in the last 1 My are not displayed in the time series (see Methods M1a).

## Discussion

This work presents a curve of worldwide MDC occurrences as a proxy for seafloor methane emission across the last 150 My. Statistical and cyclicity analyses demonstrate a significant covariance of MDC abundance with sea level and modelled OCB over the last 140 and 100 My, respectively. Sea level mainly affects smooth trends in MDC on timescales of the order of tens of My (with the main cyclicity found at 26.66 My), during which eustatic falls favoured methane seepage and potentially influenced the dynamics of methane hydrates dissociation on ocean margins^20,49,61^. Although an increase of MDC abundance can ostensibly be associated with short-term sea level drops (< 5 My duration), most of the rapid sea level variations are not associated with significant variations in MDC abundance. This observation suggests that sea level may not be the principal controller of short-term seafloor methane seepage at a global scale but combines with other factors in regulating a complex and dynamic global seepage system. For example, glacial periods, which are commonly characterised by fluctuations of sea level, are potentially more favourable to record an increase in methane seepage. However, this effect may be balanced by the contemporaneous expansion of cooler bottom waters and thus enhanced gas hydrate formation, which lead to an overall reduction of seafloor seepage^73–75^. On the contrary, isostatic rebound during higher sea level can induce methane release^76^.

The association of MDC formation and the deposition of organic-rich sediments has been recognised locally^77^ but has not been demonstrated before in the globally distributed data and at the long temporal scale of our compilation. The modelled OCB curve we employ shows 0 My and ~60 My correlation time-lags with MDC occurrence (Fig. 3c), and also shows the same cyclicities detected in the MDC record in data younger than 70 Ma (Fig. S8). The OCB reflects organic matter accumulation in the sedimentary column and thus potential hydrocarbon generation. To better understand the observed time-lags between OCB and the MDC we need to consider the processes leading to methane generation, which oxidation favours the MDC formation. The time-lag maximum at 0 My suggests that biogenic methane, generated from newly deposited organic matter, is the principal source of the methane seeping at the seafloor during the last 100 My. Therefore, OCB likely represents a parameter that instantly influences seafloor methane emission over geologically very short (i.e. <1 My) periods because the generation of biogenic methane is a geologically instantaneous process (resolved as zero time-lag in our curve), relative to the time scale for formation of MDC. Biogenic methane generation is active at the low temperatures (<50 °C) typical of shallow burial and numerous gas accumulations show that sizable volumes of biogenic methane can be generated within short geological time intervals^78^. In contrast, thermogenic methane is commonly documented at seepage sites where deep hydrocarbon source rocks and reservoirs are connected to the surface via structural discontinuities (e.g. faults) or carrier beds^79^. The generation of thermogenic methane normally requires the burial of organic matter at depth until suitable temperatures (>50–60 °C) are reached for thermal cracking of kerogens. The generated gas then must be expelled from the source rock and migrate towards the surface. The entire process, from organic matter deposition to thermogenic methane seepage, requires tens of My^80^ and thus is suggested for the correlation between OCB and MDC at a time-lag of ~60 My. As such, we suggest that this time-lag reflects the signal of thermogenic methane specifically, and thermogenic methane acts as secondary contributor to the seafloor methane budget. However, it must be considered that the 100 My-length of the OCB time series limits the significance of the~ 60 My observation.

The two prominent cyclicities in MDC occurrence recognised by spectral analysis (C1 = 1/26.66 My^−1^ and C2 = 1/12 My^−1^) are also identified in the sea level and OCB data. However, in detail, sea level controls the lowest frequency cyclicity in MDC from 150 My, whilst OCB becomes the most significant cyclic controller at the end of the Late Cretaceous at ~70 Ma. This interpretation is linked to the limited extent of the OCB time series to 100 My, and the role of OCB can be hypothesized to extend further back in time.

The dynamics of methane escape to the global ocean undoubtedly involve interrelating additional factors, such as climate, sediment input and plate tectonics, not explicitly considered here. The fundamental MDC cyclicity found in this work (with a period of ~27 My) is strikingly similar to the 26 My cyclicities observed, for example, in the carbon cycle associated with plate tectonic processes^81^, in atmospheric CO_2_^81^, in oceanic anoxic events^82^, and in mass extinction events^82,83^. Additionally, the C2 cycle (12 My period) is close to the ~9 Myr cyclicity identified in Cenozoic/Mesozoic foraminifera δ^18^O^84^, carbon-cycle^85^ and sedimentological proxies^86^. The origin of this cyclicity has been attributed to both the modulation of Milankovitch eccentricity cycles and variations in global tectonics^84,85^.

These analogies show that the proposed reconstruction of seafloor methane seepage across the last 150 My is related to a large spectrum of global phenomena, and thus has key implications for a better understanding of methane cycling at the present day. Notably, the modern expansion of hypoxic zones in marine shelf environments^87,88^ with the resulting increase in OCB may lead to an increase in seawater methane concentration over the coming centuries. Recent work has cast doubt on whether such seafloor emissions will lead to a net increase in greenhouse gases^89^, but taken together our results emphasise the importance of seafloor methane leakage as a critical but hitherto underappreciated component of the global carbon cycle.

## Methods

### M1 - Building and characterising a MDC record based on dated methane seepage samples

#### a) Building the record and testing its robustness

We compiled an original dataset of the documented occurrences of MDC up to 150 Ma by implementing similar compilations available in literature^20,90^. We counted as a single entry in the dataset any formation hosting MDC in a defined sedimentary basin. When a formation name is not available, MDC falling within an arbitrary 100 km radius within the same basin have been considered as a single entry. The minimum and maximum ages of dating for each carbonate sample have been determined either by radiometric dating (where available) or by considering the age of the hosting rock/sediment. The global MDC record we derived from it is a time series with a sampling time 1 My. Of 190 occurrences, 52 were dated in the last million years. Whilst these represent a sufficient dataset to observe variations in this time span with higher resolution sampling, our focus is on connecting modern seepage emission with those recorded across the longest available time history. We thus focused on the dataset of 138 samples dated between 1 Ma and 150 Ma. To build the record, we binned the ages for each sample in intervals of 1 My. For example, a sample dated between 2.6 and 3.5 Ma will be included in both the 2 Ma and 3 Ma age bins. A second sample dated between 3.5 and 5.2 Ma will be included in the 3 Ma, 4 Ma, and 5 Ma intervals. This binning strategy reduces the influence of samples with higher uncertainties (e.g., dated between 1 Ma and 10 Ma) on our results, so that the main variations will be due to samples dated exactly inside a time interval. The MDC occurrence record across the last 150 My is obtained by counting the occurrences in each interval (Fig. 2).

We estimated the robustness of the record for MDC based on the starting dataset, e.g., how independent it is of the particular selection of data. We tested the statistical robustness of the dataset using a bootstrapping approach (n value = 1000), as it is independent of the underlying statistical distribution of the data. We subtracted 30% of the MDC entries from the original dataset randomly and computed the normalised correlation coefficient between the original and the reduced time series. We repeated the procedure 1000 times and computed the average coefficient and average time-series over all iterations (Fig. 3a). We conclude that the dataset is stable based on the high average correlation coefficient (0.9605) and the minimal variance (twice the standard deviation = 0.0025) of the 30% bootstrap test (Fig. 3a). The correlation coefficient follows a normal distribution with these mean and standard deviation, as shown by a Kolmogorov-Smirnov test using a 5% significance threshold.

#### b) Assessing instability and removing biasing trends from the record

The MDC record shows important temporal trends that can be modelled (i.e. fitted) using polynomial and sinusoidal regressions. The samples that can be collected are likely more representative of modern than ancient times. Such a difference is likely causing the higher variability of MDC in modern times (Fig. 2). We performed linear, quadratic, cubic and sinusoidal regressions (Supplementary Fig. S3) and plotted the corresponding residuals: here, one or more quasi-periodic trends appear in all residuals, which are not stationary across the last 150 My. This inference is confirmed by a Durbin-Watson hypothesis test performed for each regression: the test outputs zero if the null hypothesis of stationarity in the residual is rejected, i.e., there is absence of autocorrelation among residuals at lag 1. The null hypothesis of absence of autocorrelation among the residuals is rejected at the 5% significance level in all cases (the *p*-value is 10^−5^ or lower). We selected a linear (decreasing) regression with time over the other regressions as the residual reduction for regression models of higher grade or complexity does not decrease sufficiently to justify their application^91^.

#### c) Spectral analysis, characterization and reconstruction of the MDC record from cyclicities

A strong perturbation may rule the deterministic behaviour of the linear residuals and create cyclicity; such signals can be recognised using matched filters, then calculating the spectrogram of the filtered signal^92,93^. As we do not have a reference to build a template-wavelet for matched filtering, we used the simplest approach, which is filtering the linear residuals using as filter coefficients the signal time-reversed^93^. We obtained the spectrogram of the filtered residuals with Hamming windows of 5 My and 60% overlap^92^ (Supplementary Fig. S4, threshold at 145 dB/My). These parameters allow the reconstruction of peaks while retaining stability. Two signals involve cyclicities with periods shorter than 4 My^−1^ (dashed white line – this is the limit cyclicity we decided to interpret given a 1 My sampling). They span the 6–21 My and 50–70 My intervals: the two intervals will be discussed in terms of its inherent cyclicities and amplitude variations. We observe that the most important cyclicity retrieved in these two periods is C2 = 1/12 My^−1^.

In addition to the matched filtering (Fig. S4), we studied cyclicities by computing the spectrograms of the MDC residuals (Supplementary Fig. S5a,b). The spectrograms are computed using Hamming windows of 10 My and 5 My (60% overlap, threshold at 140 dB/My^−1^) as the variations in windowing allow us to estimate lower and higher frequencies across the signal. For a 10 My window, we estimate a second median cyclicity (red circles – Supplementary Fig. S5b) as C1 = 1/26.66 My^−1^ ± 45%, where the uncertainty is given by the measurements’ variance. For a 5 My window the spectrograms confirm the onset of a median cyclicity of C2 = 1/12 My^−1^ ± 20% (Supplementary Fig. S5a) obtained with matched filtering. C1 and C2 stabilize during periods of strong MDC perturbations (50–70 Ma and 6–21 Ma) and become unstable, or disappear, for smaller MDC variations. We note that while C1 is more likely to dominate the entire 0–150 My time span given its lower sensitivity to shorter variations in the MDC controller, C2 is the clearest and most stable cyclicity after 49 My, as already observed using the matched filter. In the following analysis, we discuss the different spectral behaviours in the intervals 1–49 Ma, 50–70 Ma, and 71–150 Ma (Supplementary Fig. S5a, vertical dashed lines).

The full power spectrum of our MDC data across the last 150 My is obtained after filtering out all cyclicities of higher frequency than 1/50 My^−1^ (Supplementary Fig. S6, middle raw). This confirms that C1 and C2 dominate the MDC record. By calculating the power and phase spectra (Supplementary Fig. S6a,b) across the 1–70 Ma and 71–150 Ma time periods we observe that C1 (red vertical line) is effectively a stable cyclicity acting between 1 and 150 Ma, even if for the most recent period only two C1 cycles could be reconstructed. C2 (black line) is, in contrast, absent before 70 Ma. However, after this date, C2 becomes an important controller of MDC cyclicity (amplitude is > 1/2 of that of C1).

We want to see to what extent the entire MDC residual across the last 150 My can be reconstructed from the amplitudes and phases obtained using the spectral analysis only. The analytic signal derived from these parameters (frequencies, amplitudes and phases) reconstructs most of the filtered MDC residual variations (Fig. 4). The fit between C1 and MDC before 70 My is excellent, except for a transient in the period 130–140 Ma (r^2^ = 0.56). The agreement between the record and model worsens between 0 and 70 My ago (r^2^ = 0.12) comprising both a period of amplitude decrease (50–70 Ma) and one of strong variations (40–49 Ma). By adding cyclicity C2, the agreement between record and model is partially restored between 0 and 40 Ma (r^2^ = 0.36).

### M2 - Stationary comparison with alternative observations

This work compares the MDC record with its potential geological driver(s) on the various intervals covered by the other investigated time series. We obtained representative time series of: seawater sulfate^59^, global sea level variation^21–23^, deep-sea temperature^24^, modelled Organic Carbon Burial (OCB)^25^, and sediment accumulation (SV)^60^. We re-sampled all the corresponding time series at 1 My and consider them as a unique ensemble.

We used Principal Component Analysis to discriminate meaningful dependency, i.e. observations that are (or are not) strictly correlated and add to the total variance. The analysis is on data after linear detrending. Three principal components account for most of the variability in the ensemble ( >85% - PC1 = 48%; PC2 = 20%; PC3 = 11%). The most important observations are (Fig. 3b; Supplementary Fig. S2):

1. PC1 defines three groups of data (Figs. 3b and S2, left): (1) MDC and Sulfates (positively correlated); (2) OCB, SV, and sea level from Müller *et al*. (2008); (3) the two remaining sea levels and temperature (negatively correlated). More generally, SV only provides a significant contribution to PC3 (Supplementary Fig. S2, left).
2. Different sea level models^21–23^ correspond to different variations for PC1, which accounts for 48% of the variations in our dataset - the corrected Miller *et al*. 2005 curve shows the highest anticorrelation for both PC1 and PC2;
3. Sea level and deep-sea temperature are positively correlated (r^2^ = 0.48) and give similar contributions to PC1.

Considering the different contributions of the components to variability (PC3~1/5 PC1 and ~1/2 PC2) we decided to: (1) consider the corrected sea level curve only; (2) discard the sediment volumes from further analyses as they show correlations similar to those of OCB; (3) discard the temperature, which is worse-constrained than other parameters in time and highly correlated to the corrected sea level. While the corrected sea level shows significant anti-correlation with MDC (r^2^ = 0.34), sulfate and OCB show positive (but statistically insignificant - r^2^ of 0.12 and 0.09, respectively) correlation with the MDC.

We performed a second Principal Component Analysis to assess the dependence of our inferences on the number of observations included. This time, we only added to MDC the three different observations representing the main variability in the first analysis: MDC, the corrected sea level curve (anticorrelation for PCA), OCB and sulfate (positively correlated for PCA). The first two components account for more than 79% of the variance (Supplementary Fig. S2): PC1 = 55.0%; PC2 = 24.0%; PC3 = 11.87%. The PC1-PC2 plot (right) confirms the strong anticorrelation between MDC and sea level; however, from this graph there is no evidence of a preferential correlation between MDC and OCB or sulfate. This test confirms that the main controllers of MDC in the last 100 My have to be searched between sea level, sulfate and OCB.

The single oscillatory low-frequency signal observed for MDC also underlies the other three records (Fig. S7). These significant (r^2^ always > 0.4) signals have slightly different periods, (MDC – 74.26 My; sea level – 71.81 My; OCB – 62.5 My; sulfate – 69.93), with MDC having the same phase of OCB and sulfate, and the opposite phase of sea level. As we aim to obtain at least two cycles in the 100 My span, all the time series were thus filtered below 1/50 My^−1^. If this signal is removed, it is evident that the detrended sulfate residuals (Fig. S7) shows no cycle, a feature already apparent in the sulfate curve (Fig. 2): this low-period cyclicity in sulfate is due to their drastic increase especially between 50 and 40 My, the only non-linear variation in the curve in the last 100 My. The 40–50 My time span corresponds to the time of the anomalous transient in the analytic signal reconstructed from cyclicities only (Fig. 4). It thus highlights the central role of sulfate in triggering major changes in the cyclic behaviour of MDC. While the progressive increase in sulfate between 120 and 50 My might contribute to the strong changes in amplitude variations across the 50–70 My period (Figs S4–S5), the cyclic characteristics of MDC cannot be reconstructed in sulfate.

The power spectral analyses performed on the MDC were thus only applied to the sea level (in the period 0–140 Ma) and OCB (0–100 Ma) records. Supplementary Fig. S8 shows that, before 71 My ago, C1 is the only spike in the sea level spectrum and MDC records. The agreement between MDC and sea level spectra in this time span is particularly strong; we deduce that sea level change is the most significant low-frequency controller of MDC. On the other hand, C1 and C2 are the most relevant cyclicities after 70 Ma only for of MDC and OCB records. Among cyclic observations (thus excluding sulfate) the time correlation analysis described in the main text (Fig. 3c) ranks OCB as the only controllers showing instantaneous correlation. After 70 Ma ago, OCB saturates the MDC record and results a significant instantaneous controller of MDC emissions.

## Supplementary information

Supplementary Information.
