# Supplementary material for: A record of seafloor methane seepage across the last 150 million years
Source: Sci Rep. 2020 Feb 13;10:2562. doi: 10.1038/s41598-020-59431-3 (PMC7018728; doi:10.1038/s41598-020-59431-3)
Supplement: Supplementary file 1 — Supplementary Information. [file 41598_2020_59431_MOESM1_ESM.pdf]

Supplementary Material

A record of seafloor methane seepage across the last 150 million years

D. Oppo[1]*, L. De Siena[2], and D. B. Kemp[3]

[1]*School of Geosciences, University of Louisiana at Lafayette, Lafayette, LA 70503, USA.*

[2] *Institute of Geosciences, Johannes Gutenberg University Mainz, D-55128 Mainz, Germany.*

[3]*School of Earth Sciences and State Key Laboratory of Biogeology and Environmental Geology, China University of Geosciences, Wuhan 430074, China.*

**davide.oppo@louisiana.edu*

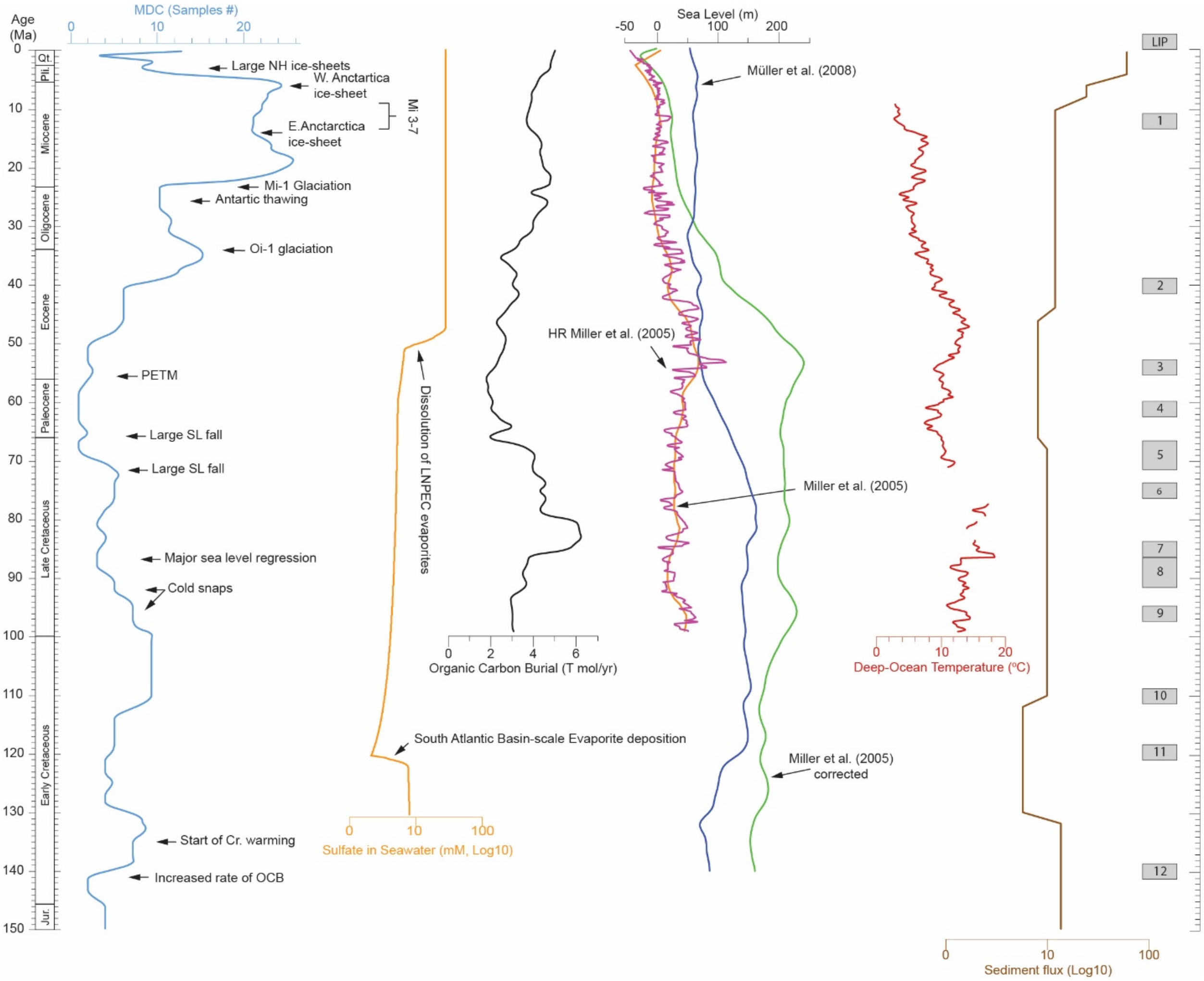

Fig. S1

Time series of methane-derived carbonates during the last 150 My. Records of Organic Carbon Burial (OCB), global sea level, deep-sea water temperature, seawater sulfate, sediment flux, and large igneous provinces (LIP) are also shown for comparison. The main climatic events are shown. References for the various time series are in the main text.

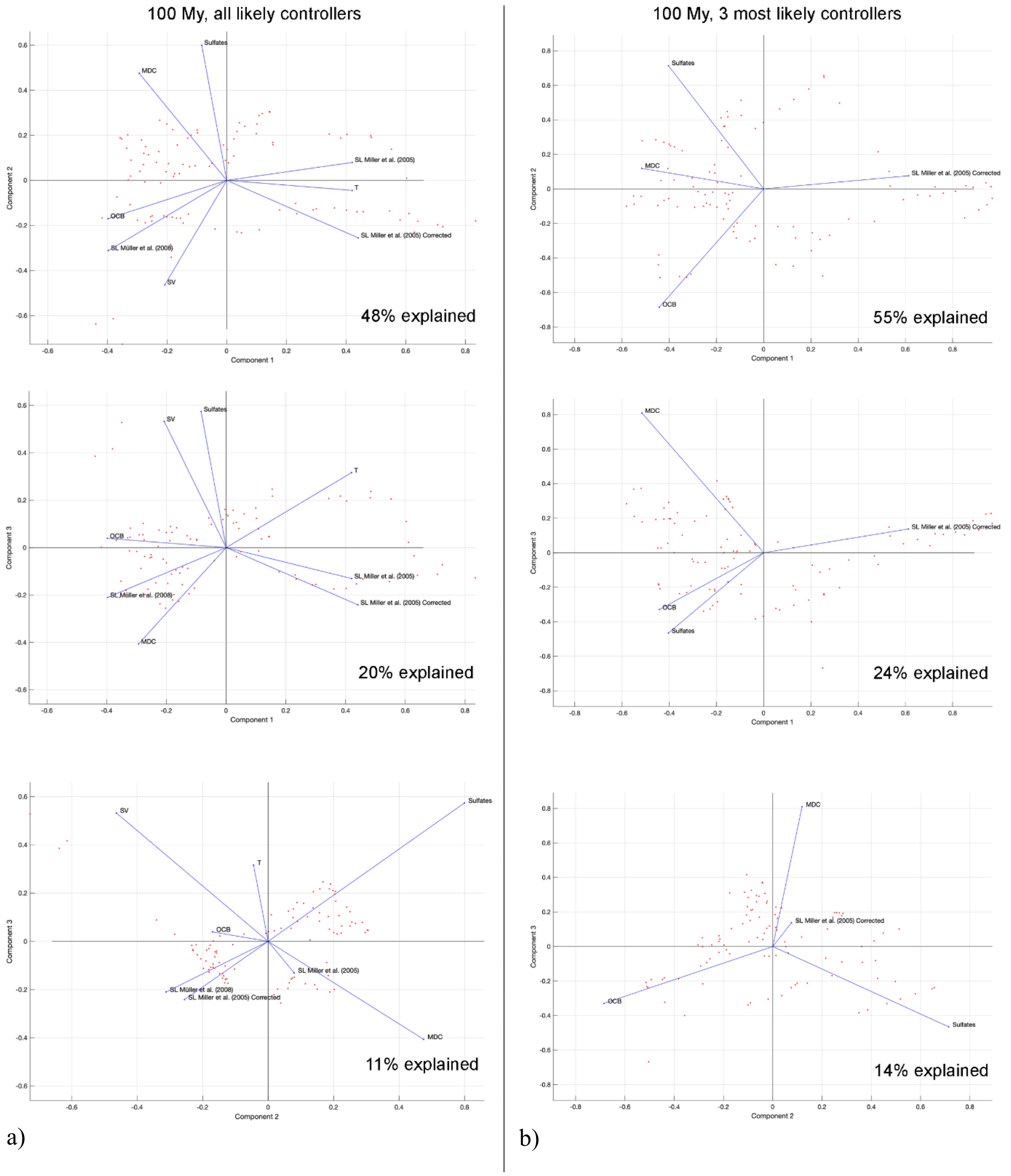

Fig. S2: Principal Component analysis. a) Principal component analysis performed on original data without regressions, on the ensemble of available measurements and models. The panels show the contribution of each observation in the two main planes defined by the first three components. These account for more than 79% of the total variance (PC1=48%; PC2=20%; PC3=11%). SV: sediment volume, SL: sea level, T: temperature, OCB: organic carbon burial, MDC: methane derived carbonate. b) Second Principal Component Analysis to assess the dependence of our inferences on the number of observations included. In this case we only consider the 4 most relevant observations.

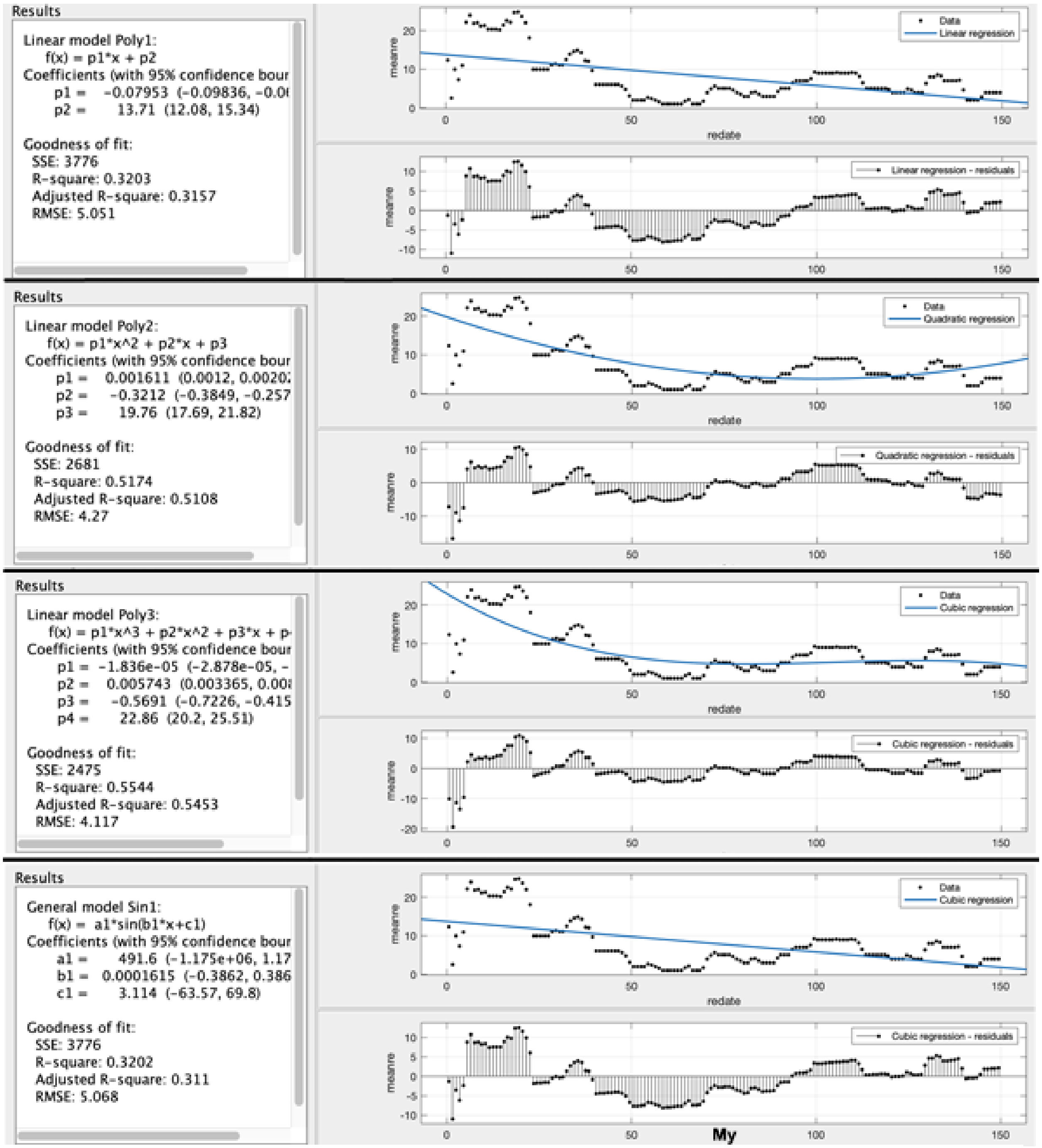

Fig. S3: **Linear, quadratic, cubic and sinusoidal regressions** with residuals.

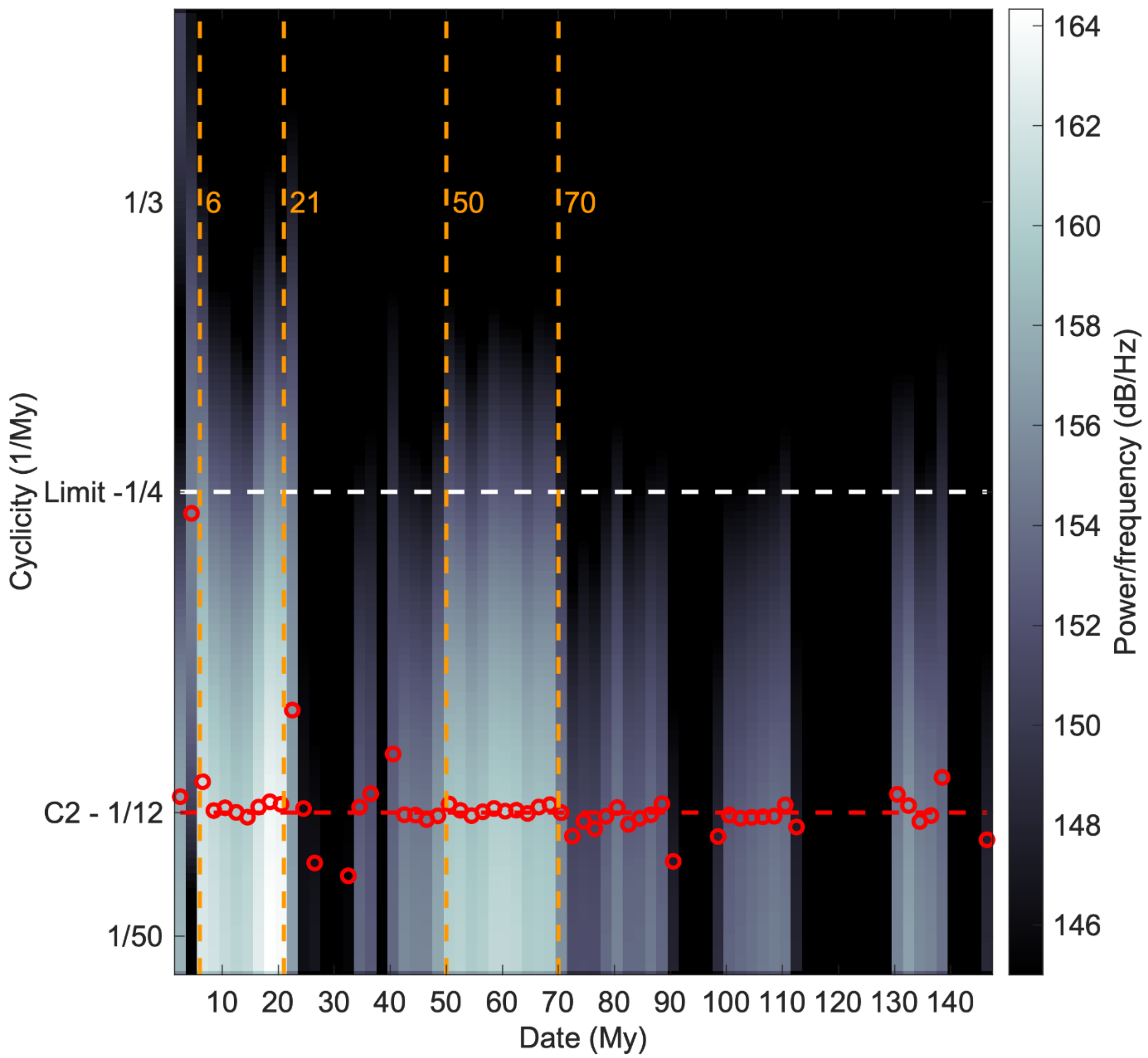

Fig. S4: **Matched filtering of the MDC residuals** using a Hamming windows of 5 My and 60% overlap. Threshold at 145 dB/My. The orange dashed lines indicate the period of the strongest MDC variations, characterise by the onset of a 1/12 cyclicity (C2 – red dashed line following the median across existing signals).

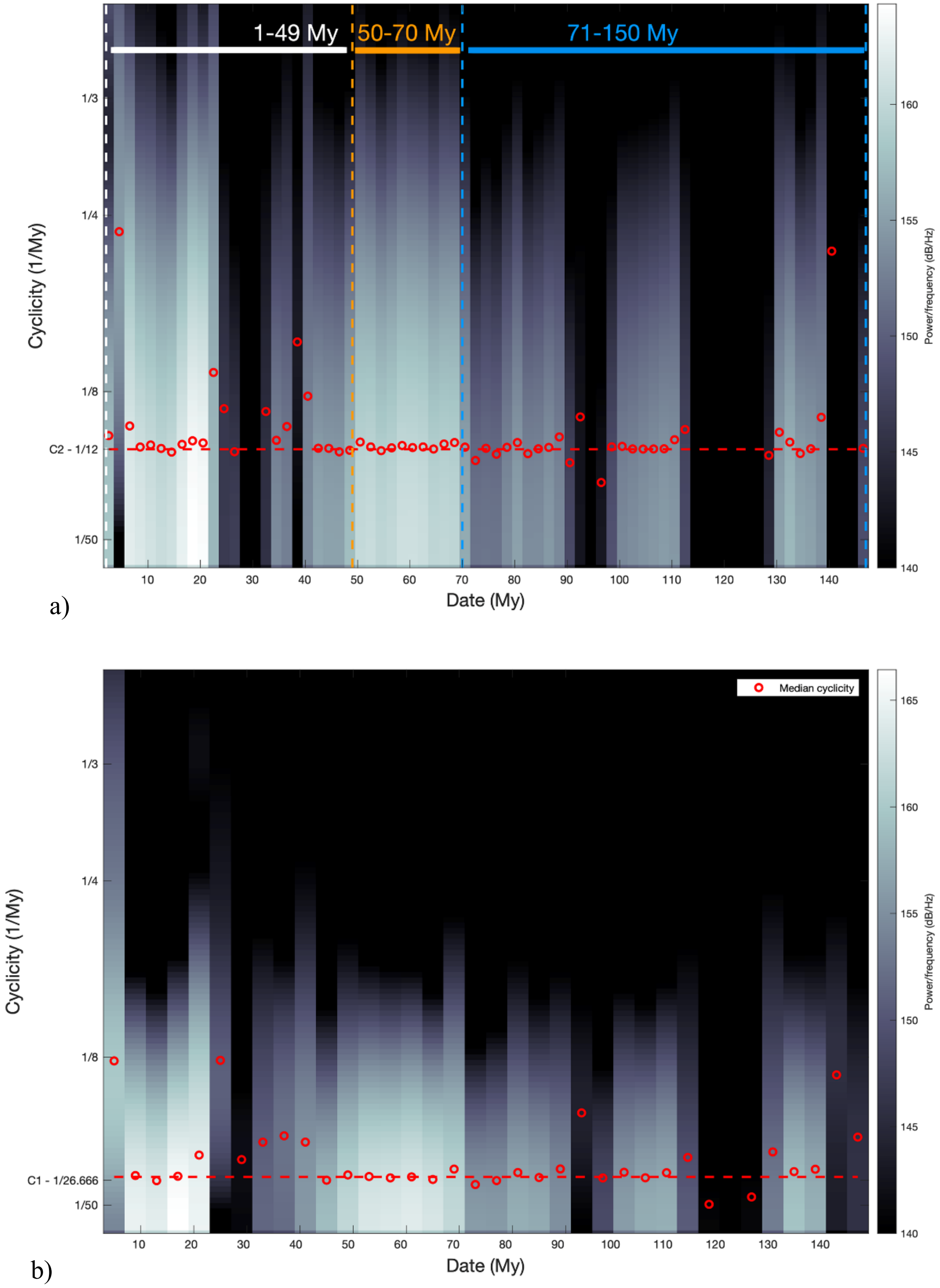

Fig. S5: **Spectrograms of the MDC residuals** computed using Hamming windows of 10 My and 5 My (60% overlap, threshold at 145 dB/My^{-1}). Red circles are median cyclicities. a) 5 My window again corresponding to median cyclicity C2; b) 10 My window corresponding to median cyclicity C1.

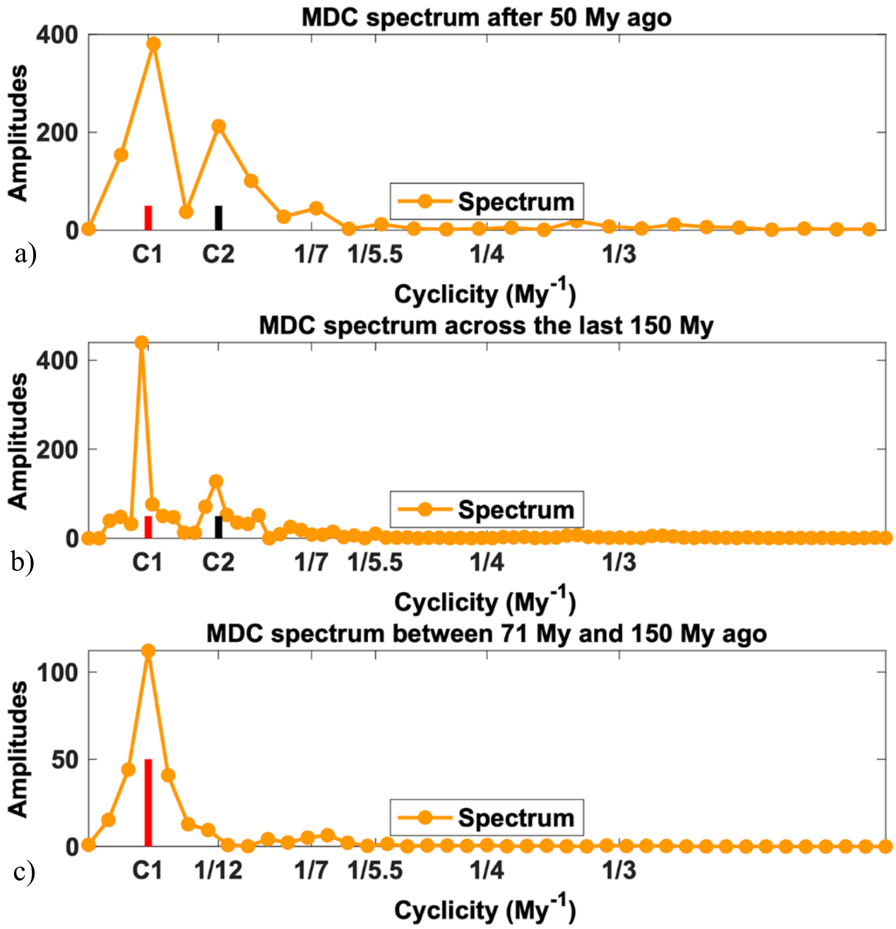

Fig. S6: Power spectrum of the MDC high-pass filtered residuals across the periods 1-49 Ma (a), 1-150 Ma (b), and 71-150 Ma (c). Cyclicity C2 does not appear in the bottom panel.

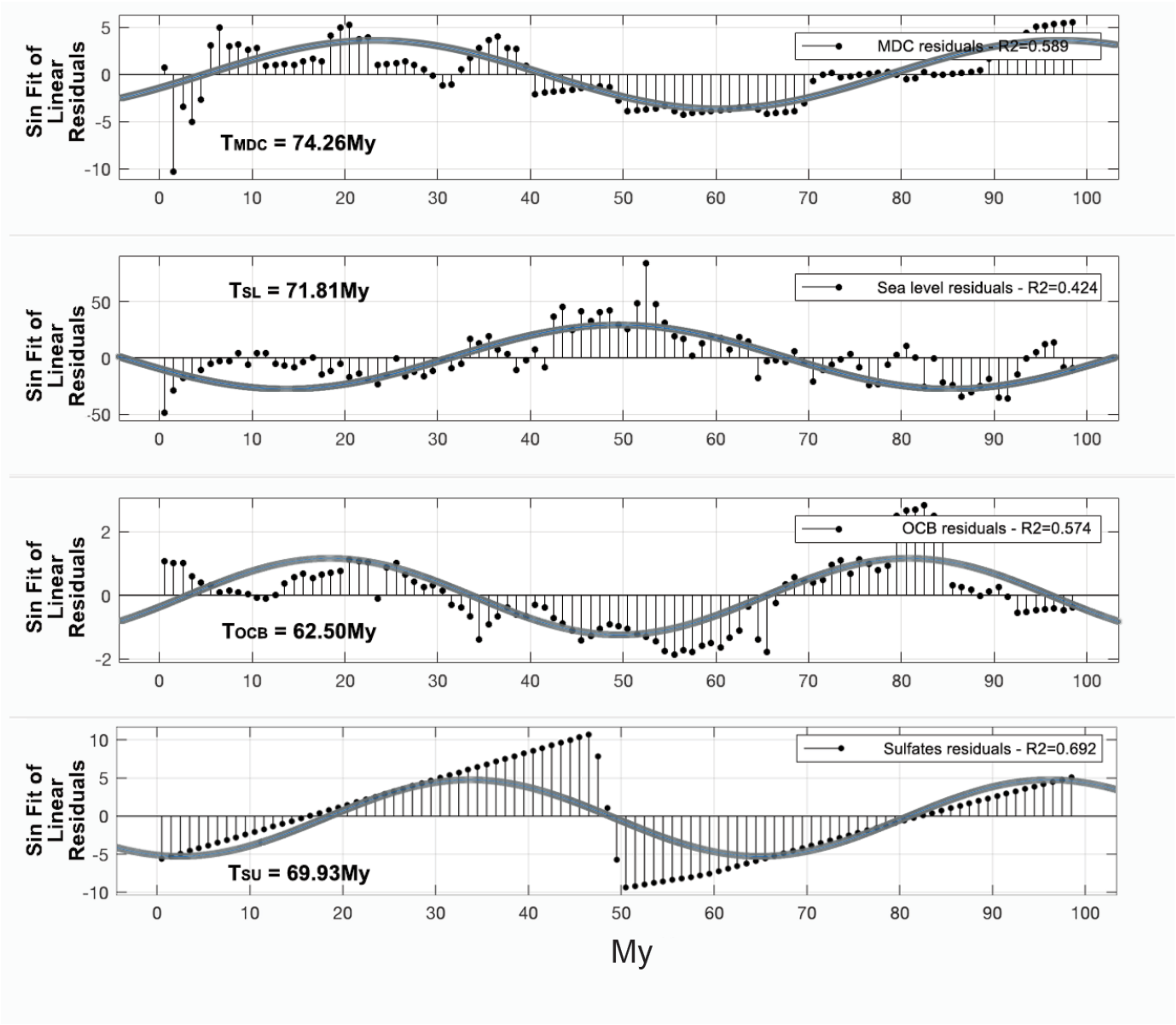

Fig. S7: Similar oscillatory signals characterising the linearly detrended MDC, sea-level, OCB and sulfates records.

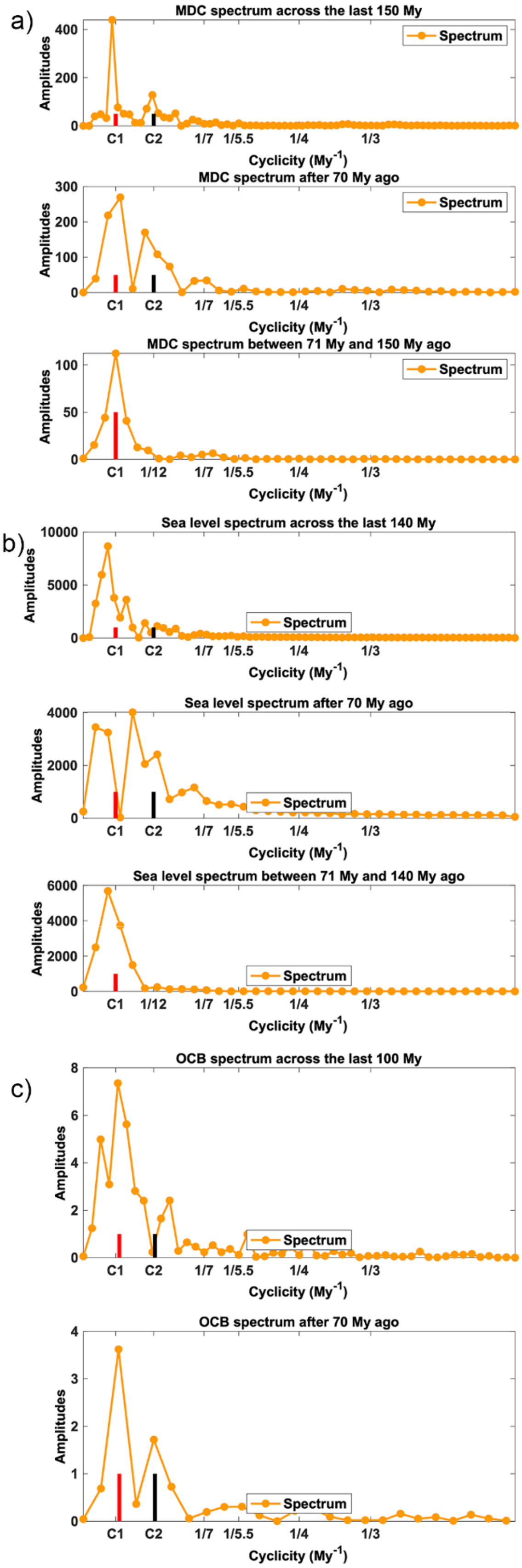

a)
MDC spectrum across the last 150 My
Amplitudes
400
200
0
Spectrum
C1
C2
1/7
1/5.5
1/4
1/3
Cyclicity (My-1)
MDC spectrum after 70 My ago
300
200
100
MDC spectrum between 71 My and 150 My ago
100
50
1/12
b)
Sea level spectrum across the last 140 My
10000
5000
Sea level spectrum after 70 My ago
4000
2000
Sea level spectrum between 71 My and 140 My ago
6000
c)
OCB spectrum across the last 100 My
8
6
4
2
OCB spectrum after 70 My ago
3
1

Fig. S8: **Power spectrum of the MDC (a), sea-level (b) and OCB (c) high-pass filtered residuals** across the periods: (a) 1-150 Ma (top), 1-70 Ma (centre), and 71-150 Ma (bottom); (b) 1-140 Ma (top), 1-70 Ma (centre), and 71-140 Ma (bottom); (c) 1-100 Ma (top), 1-70 Ma (bottom).

Supplementary Table 1. Seep-bearing Formations or equivalent

Age interval (Ma)	Formation name and/or location	Country/Location	Reference
0-0.120	Nile DSF	Mediterranean Sea	1
0-0.015	Tekirdag, Central & Cinarcik Basins, Marmara Sea	Turkey	2
0-0.120	Viking graben	North Sea	3
0.002-0.017	Offshore Norwegian margin	Norway	4
0.001-0.015	AT 340 site, Gulf of Mexico	USA	5
0.001-0.053	Alaminos Canyon, Gulf of Mexico	USA	5,6
0.001-0.005	Hydrate Ridge	USA	7
0.001-0.045	Green Canyon, Gulf of Mexico	USA	8
0.002-0.012	Hikurangi Margin	New Zealand	9
0.003-0.008	Offshore Svalbard	Svalbard	10
0.001-0.14	South China Sea	China	11
0.003-0.045	Hydrate Hole, offshore Congo Fan	Congo	12
0.001-0.005	Nile DSF	Mediterranean Sea	13
0.007-0.012	Amon mud vocano, NDSF	Mediterranean Sea	14
0.007-0.260	Hydrate Ridge	USA	7
0-0.010	Barkley Canyon	Canada	15
0.010-0.035	Offshore Joetsu	Japan	16
0.010-0.040	Offshore Noto Peninsula	Japan	17
0.015-0.019	Makran	Pakistan	18
0.021-0.023	Diapir field, offshore Congo Fan	Congo	12
0.030-0.060	Shenu area, South China Sea	China	19
0.032-0.033	Otago Peninusula	New Zealand	20
0.060-0.080	Offshore Gabon and Congo	W Africa	21
0-0.070	Mentawai fault zone	Java	22
0.001-0.047	Snail Hill, Sunda Arc	Java	22
0.250-0.360	Gulf of Cadiz	Portugal/Spain	23
0.137-0.143	Campos Basin	Brazil	24
0.250-0.270	Offshore Montenegro	Adriatic Sea	25
0.152-0.330	NE Dongsha	South China Sea	26
0.063-0.077	Shenhu	South China Sea	26
0.126-0.78	Kakinokidai Fm., Chiba Prefecture	Japan	27
0.118-0.138	Eel River Basin	USA	28
0-1	Monterey Bay	USA	29,30
0-1	Santa Barbara Basin	USA	29
0-1	Tommeliten	North Sea	29
0-1	Sea of Okhotsk	Russia	29
0-1	Kodiak Trench	USA	30
0-1	Nyegga area, Voring Basin	Norway	31
0-1	Codling Fault Zone	Irish Sea	32
0-1	Nile DSF	Mediterranean Sea	33

0-1	Offshore, St. Lawrence Estuary	Canada	34
0-1	Amsterdam mud volcano	Mediterranean Sea	35
0-1	Athina mud volcano	Mediterranean Sea	35
0-1	Offshore Costa Rica	Costa Rica	36
0-1	Bonaccia Gas Field	Adriatic Sea	37
0-1	Offshore eastern Aleutians	USA	38
0-1	Guaymas Basin	Mexico	39
0-1	Florida escarpment	USA	40
0-1	Makran accretionary prism	Pakistan	41
0-1	Outer Marianna Trough		42,43
0-1	Offshore Venice	Adriatic Sea	44
0-1	Krishna–Godavari Basin	India	45
0-1.8	Dnjepr canyon	Black Sea	46
1.4-1.65	Enza River, Northern Apennines	Italy	47
1.8-2.5	Koshiba Fm., Kanagawa Prefecture	Japan	27
1.8-3.6	Bata Fm	Philippines	48
1.8-23	Nadachi Fm.	Japan	49
2.5-3.6	Hijikata Fm., Shizuoka Prefecture	Japan	27
2.5-23	Quinault Fm	USA	50
2.5-3.6	Tamari & Horinouchi Fms., Shizuoka Prefecture	Japan	27
2.5-5.3	Quinault Fm; Quinault Coast	USA	51
2.5-5.3	Maarai Fm., Chiba Prefecture	Japan	27
2.5-5.3	Shiramazu Fm., Chiba Prefecture	Japan	27
2.5-5.3	Kawazume Fm., Niigata Prefecture	Japan	27
2.5-5.3	Quinault Fm., Washington	USA	15
3.6	Stirone River	Italy	52
3.6-28	Navidad Fm.	Chile	53
5-11	Santa Cruz Mudstone	USA	54
5-11	Hunghuatzu Fm., Nantzuhsien River	Taiwan	55
5-16	Montardone melange, N. Apennines	Italy	56
5-23	Columbia River, Washington	USA	57
5.3-11.6	Nodani Fm.	Japan	58
5.3-23	Taira Fm	Japan	59
5.3-23	Ogaya Fm.	Japan	60
5.3-23	Bessho Fm	Japan	60
5.3-23	Bexhaven Limenstone	New Zealand	61
5.3-23	Hiranita Fm.	Japan	62
5.3-33.9	Sagavanirktok Fm., North Slope Alaska	USA	63
5.3-11.6	Nodani Fm., niigata Prefecture	Japan	27
5.3-11.6	Morai Fm., Central Hokkaido	Japan	27
5.3-23.3	Moonlight limestone; NE Coastal basin Northern Island	New Zealand	64
5.3-23.3	Marmorito, Monferrato	Italy	65
5.3-7.2	Verrua Savoia, Tertiary Piedimont Basin	Italy	66

5.3-7.2	Ripa dello Zolfo, Tertiary Piedimont Basin	Italy	66
5.3-7.2	Calcare Solfifero, Sicily	Italy	67
5.4	Akaishi Fm., Fukaura Town	Japan	68
5.5-9.3	Perto Escondido	Venezuela	69
6-8	Lucina limestone	Italy	70
6-11.1	Godineau River	Trinidad	69
6.4-10	Jordan Hill	Trinidad	69
6.65-21.25	Caujarao	Venezuela	69
8	Lucina limestone, Pietralunga	Italy	65
8.2-9	Urenui Fm., Taranaki Basin	New Zealand	71
10-20	Hayama Fm	Japan	72
10-20	Lengua Fm	Trinidad	73
10-20	Pozon Fm	Venezuela	73
11-16	Lucina limestone	Italy	70
11.6-23	Higashibessh Fm.	Japan	74
11.6-23	Fosso Riconi	Italy	75
11.6-16	Nupinai Fm.	Japan	76
13.3-13.75	Vicchio Marls	Italy	77
15	Hayama Fm.	Japan	78
15.9-23	Setogawa Group, Shizuoka Prefecture	Japan	27
15.9-23	Aokiyama Fm., Chiba Prefecture	Japan	27
16-20.4	Kokozura Fm.	Japan	79
16-23	Muroto Fm	Japan	80
16-23	Shikiya Fm.	Japan	81
16.85-17.3	Plamar-Molinera	Colombia	69
16.85-17.3	Plamar-Molinera	Colombia	69
18.75-23.3	Bath Cliff	Barbados	69
18.5-20	Cantera portugalete	Cuba	69
18.6-21.25	Corro Colorado	Venezuela	69
20-30	Astoria Fm	USA	82
20-30	Talara Fm	Peru	83
20-30	Heath shales	Peru	83
20.5-23.1	Buena Vista de Maicillal	Venezuela	69
23-28	Pysht/Sooke Fm.,Washington	USA	15
23-28	Keasey Fm	USA	84
23-33	Krosno Fm	Poland	85
23-33	Lincoln Creek Fm	USA	86
23-33	Satsop River, Lincoln Creek	USA	57
23-33	Clatskanie, Oregon	USA	57
28-33	Makah Fm., Washington	USA	57
28-33.9	Heath shales, Belén	Peru	87
28.4-33.9	Nuibetsu Fm., Kami-Atsunai Station	Japan	68
28.4-37.2	Tanamigawa Fm., Honshu	Japan	80

30-36	Canyon River, Washington	USA	88
30-40	Diapiric melange	Barbados	89
30-40	SOFZ	Barbados	89
33.9-55.8	Poronai Fm., Central Hokkaido	Japan	27
33-37	Wagonwheel Fm	USA	83
33.9-48	West Fork of Grays River, Washington	USA	88
33-40	Humptulips Fm	USA	86
33-40	Lincoln Creek Fm	USA	86
33.9-40	Bear River, Washington	USA	88
33-40	Siltstone of Units B	USA	86
33-40	Whiskey Creek	USA	90
33-40	Humptulips Fm	USA	91
33-55	Keasey Fm., Oregon	USA	57
35-49.25	Elmira asphalt mine	Cuba	69
36.3-37.6	Joes River	Barbados	69
40-50	Central Istria Flysh	Croatia	92
40-50	Oomagari Fm	Japan	93
50	Pobiti Kamani	Bulgaria	94
55-58	Fossildalen	Svalbard	95
55-65	Moreno Fm, Panoche Hills	USA	19,96
65-66	James Ross Basin, Snow Hill Island	Antarctica	97
65-66	James Ross Basin, Seymour Island	Antarctica	88
65.5-99.6	Sada Limestone, Shikoku	Japan	98
70.3-73	Tepee Buttes	USA	99,100
70.6-83.5	Guenoc Ranch & Romero Creek	USA	101
70-80	Omagari Fm	Japan	102
72-126	Raukumara Peninsula	New Zealand	103
72.1-78	Western Interior Seaway, Montrose	USA	88
78	Whangai Fm. North Islanf	New Zealand	103
83-85	Maeshima, Kyushu	Japan	88
83-100	Shangba Section	Tibet	104
90-100	Tenkaritoge Fm	Japan	105
90-100	Kadui, Jiding, and Walie Sections	Tibet	104
93.6-99.6	Ezo Group, NW Hokkaido	Japan	27
94-100	Gamba Section	Tibet	104
99.6-112	Ezo Group, Central Hokkaido	Japan	27
99.6-125	Great Valley Group, Cold fork of Cottonwood	USA	106
99-112	Ispaster	Spain	107
100-110	Hikagenosawa Fm	Japan	108
100-113	Black Flysch Group, Basque-Cantabrian Basin	Spain	109
100-113	Ellef Ringnes Island	Canada	110
100-113	Xiege Section	Tibet	104
100-125	Christopher Fm	Canada	111

106	Yezo Group	Japan	78
110-120	Logoda Fm	USA	84
113-120	Marnes Bleues Fm., Vocontian Basin	France	112
120-130	La Perlita limestone	Mexico	84
125-130	Kuhnpasset Beds	Greenlalnd	113
125-130	Hradiste Fm., Baska	Czech Republic	114
125-134	Sinaia Fm, Carpatian Mountains	Romania	115
130-133.9	Great Valley Group, Wilburg Spring & Rice Valley	USA	106
130-134	Planerskoje section	Ukraine	116
130-140	Chebo Fm	China	84
130-140	Maiolica Fm	Italy	84
130-140	Sangxiu Fm	China	84
130-140	Upper Grodziszcze Fm	Czech Republic	84
130-134	Curnier, Rottier	France	117
133.9-140	Great Valley Group, Rocky & Bear Creeks	USA	118
133-140	Crack Canyon Fm, Rocky Creek	USA	118
134-139	Sandy limestone, Novaya Zemlya	Russia	119
139-152	Sassenfjorden	Svalbard	120
140-150	Stony Creek Fm	USA	84
145	Black Limetone, Novaya Zemlya	Russia	119
145.5-150.8	Great Valley Group, Paskenta	USA	106
145-150	Gateway Pass beds	Antarctica	121

1. Pierre, C., Bayon, G., Blanc-Valleron, M.-M., Mascle, J. & Dupré, S. Authigenic carbonates related to active seepage of methane-rich hot brines at the Cheops mud volcano, Menes caldera (Nile deep-sea fan, eastern Mediterranean Sea). *Geo-Mar. Lett.* **34**, 253–267 (2014).
2. Crémière, A. *et al.* Methane-derived authigenic carbonates along the North Anatolian fault system in the Sea of Marmara (Turkey). *Deep Sea Res. Part Oceanogr. Res. Pap.* **66**, 114–130 (2012).
3. Hovland, M., Talbot, M. R., Qvale, H., Olaussen, S. & Aasberg, L. Methane-related carbonate cements in pockmarks of the North Sea. *J. Sediment. Res.* **57**, 881–892 (1987).
4. Crémière, A. *et al.* Fluid source and methane-related diagenetic processes recorded in cold seep carbonates from the Alvheim channel, central North Sea. *Chem. Geol.* **432**, 16–33 (2016).
5. Feng, D. *et al.* U/Th dating of cold-seep carbonates: An initial comparison. *Deep Sea Res. Part II Top. Stud. Oceanogr.* **57**, 2055–2060 (2010).
6. Aharon, P., Schwarcz, H. P. & Roberts, H. H. Radiometric dating of submarine hydrocarbon seeps in the Gulf of Mexico. *Geol. Soc. Am. Bull.* 12 (1997).
7. Teichert, B. M. A. *et al.* U/Th Systematics and ages of authigenic carbonates from Hydrate Ridge, Cascadia Margin: Recorders of fluid flow variations. *Geochim. Cosmochim. Acta* **67**, 3845–3857 (2003).
8. Bian, Y., Feng, D., Roberts, H. H. & Chen, D. Tracing the evolution of seep fluids from authigenic carbonates: Green Canyon, northern Gulf of Mexico. *Mar. Pet. Geol.* **44**, 71–81 (2013).

9. Liebetrau, V., Eisenhauer, A. & Linke, P. Cold seep carbonates and associated cold-water corals at the Hikurangi Margin, New Zealand: New insights into fluid pathways, growth structures and geochronology. *Mar. Geol.* **272**, 307–318 (2010).
10. Berndt, C. *et al.* Temporal Constraints on Hydrate-Controlled Methane Seepage off Svalbard. *Science* **343**, 284–287 (2014).
11. Han, X., Suess, E., Liebetrau, V., Eisenhauer, A. & Huang, Y. Past methane release events and environmental conditions at the upper continental slope of the South China Sea: constraints by seep carbonates. *Int. J. Earth Sci.* **103**, 1873–1887 (2014).
12. Feng, D., Chen, D., Peckmann, J. & Bohrmann, G. Authigenic carbonates from methane seeps of the northern Congo fan: Microbial formation mechanism. *Mar. Pet. Geol.* **27**, 748–756 (2010).
13. Bayon, G., Henderson, G. M. & Bohn, M. U–Th stratigraphy of a cold seep carbonate crust. *Chem. Geol.* **260**, 47–56 (2009).
14. Bayon, G. *et al.* Formation of carbonate chimneys in the Mediterranean Sea linked to deep-water oxygen depletion. *Nat. Geosci.* **6**, 755–760 (2013).
15. Joseph, C. *et al.* Using the 87Sr/86Sr of modern and paleoseep carbonates from northern Cascadia to link modern fluid flow to the past. *Chem. Geol.* **334**, 122–130 (2012).
16. Watanabe, Y., Nakai, S., Hiruta, A., Matsumoto, R. & Yoshida, K. U–Th dating of carbonate nodules from methane seeps off Joetsu, Eastern Margin of Japan Sea. *Earth Planet. Sci. Lett.* **272**, 89–96 (2008).
17. Hiruta, A., Wang, L.-C., Ishizaki, O. & Matsumoto, R. Last glacial emplacement of methane-derived authigenic carbonates in the Sea of Japan constrained by diatom assemblage, carbon-14, and carbonate content. *Mar. Pet. Geol.* **56**, 51–62 (2014).
18. Himmler, T. *et al.* Formation of seep carbonates along the Makran convergent margin, northern Arabian Sea and a molecular and isotopic approach to constrain the carbon isotopic composition of parent methane. *Chem. Geol.* **415**, 102–117 (2015).
19. Ge, L. & Jiang, S.-Y. Sr isotopic compositions of cold seep carbonates from the South China Sea and the Panoche Hills (California, USA) and their significance in palaeooceanography. *J. Asian Earth Sci.* **65**, 34–41 (2013).
20. Orpin, A. R. Dolomite chimneys as possible evidence of coastal fluid expulsion, uppermost Otago continental slope, southern New Zealand. *Mar. Geol.* **138**, 51–67 (1997).
21. Pierre, C. *et al.* Authigenic carbonates from active methane seeps offshore southwest Africa. *Geo-Mar. Lett.* **32**, 501–513 (2012).
22. Wiedicke, M. & Weiss, W. Stable carbon isotope records of carbonates tracing fossil seep activity off Indonesia. *Geochem. Geophys. Geosystems* **7** (2006).
23. Magalhães, V. H. *et al.* Formation processes of methane-derived authigenic carbonates from the Gulf of Cadiz. *Sediment. Geol.* **243–244**, 155–168 (2012).
24. Wirsig, C., Kowsmann, R. O., Miller, D. J., de Oliveira Godoy, J. M. & Mangini, A. U/Th-dating and post-depositional alteration of a cold seep carbonate chimney from the Campos Basin offshore Brazil. *Mar. Geol.* **329–331**, 24–33 (2012).
25. Angeletti, L. *et al.* The “chimney forest” of the deep Montenegrin margin, south-eastern Adriatic Sea. *Mar. Pet. Geol.* **66**, 542–554 (2015).
26. Tong, H. *et al.* Authigenic carbonates from seeps on the northern continental slope of the South China Sea: New insights into fluid sources and geochronology. *Mar. Pet. Geol.* **43**, 260–271 (2013).

27. Majima, R., Nobuhara, T. & Kitazaki, T. Review of fossil chemosynthetic assemblages in Japan. *Palaeogeogr. Palaeoclimatol. Palaeoecol.* **227**, 86–123 (2005).
28. Bailey, J. V. *et al.* Pseudofossils in relict methane seep carbonates resemble endemic microbial consortia. *Palaeogeogr. Palaeoclimatol. Palaeoecol.* **285**, 131–142 (2010).
29. Naehr, T. H. *et al.* Authigenic carbonate formation at hydrocarbon seeps in continental margin sediments: A comparative study. *Deep Sea Res. Part II Top. Stud. Oceanogr.* **54**, 1268–1291 (2007).
30. Gieskes, J. *et al.* A study of the chemistry of pore fluids and authigenic carbonates in methane seep environments: Kodiak Trench, Hydrate Ridge, Monterey Bay, and Eel River Basin. *Chem. Geol.* **220**, 329–345 (2005).
31. Mazzini, A., Svensen, H., Hovland, M. & Planke, S. Comparison and implications from strikingly different authigenic carbonates in a Nyegga complex pockmark, G11, Norwegian Sea. *Mar. Geol.* **231**, 89–102 (2006).
32. O'Reilly, S. S. *et al.* Shallow water methane-derived authigenic carbonate mounds at the Codling Fault Zone, western Irish Sea. *Mar. Geol.* **357**, 139–150 (2014).
33. Römer, M. *et al.* Methane fluxes and carbonate deposits at a cold seep area of the Central Nile Deep Sea Fan, Eastern Mediterranean Sea. *Mar. Geol.* **347**, 27–42 (2014).
34. Lavoie, D., Pinet, N., Duchesne, M., Bolduc, A. & Larocque, R. Methane-derived authigenic carbonates from active hydrocarbon seeps of the St. Lawrence Estuary, Canada. *Mar. Pet. Geol.* **27**, 1262–1272 (2010).
35. Himmler, T., Brinkmann, F., Bohrmann, G. & Peckmann, J. Corrosion patterns of seep-carbonates from the eastern Mediterranean Sea: Corrosion patterns of vuggy seep-carbonates. *Terra Nova* **23**, 206–212 (2011).
36. Levin, L. A. *et al.* Biodiversity on the Rocks: Macrofauna Inhabiting Authigenic Carbonate at Costa Rica Methane Seeps. *PLOS ONE* **10**, e0131080 (2015).
37. Capozzi, Guido, F. L., Oppo, D. & Gabbianelli, G. Methane-Derived Authigenic Carbonates (MDAC) in northern-central Adriatic Sea: Relationships between reservoir and methane seepages. *Mar. Geol.* **332–334**, 174–188 (2012).
38. Suess, E. *et al.* Fluid venting in the eastern Aleutian Subduction Zone. *J. Geophys. Res. Solid Earth* **103**, 2597–2614 (1998).
39. Simoneit, B. R. T., Lonsdale, P. F., Edmond, J. M. & Shanks, W. C. Deep-water hydrocarbon seeps in Guaymas Basin, Gulf of California. *Appl. Geochem.* **5**, 41–49 (1990).
40. Paull, C. K. *et al.* Indicators of Methane-Derived Carbonates and Chemosynthetic Organic Carbon Deposits: Examples from the Florida Escarpment. *PALAIOS* **7**, 361 (1992).
41. von Rad, U. *et al.* Authigenic carbonates derived from oxidized methane vented from the Makran accretionary prism off Pakistan. *Mar. Geol.* **136**, 55–77 (1996).
42. Haggerty, J. Evidence from fluid seeps atop serpentine seamounts in the Mariana forearc: Clues for emplacement of the seamounts and their relationship to forearc tectonics. *Mar. Geol.* **102**, 293–309 (1991).
43. Okumura, T. *et al.* Brucite chimney formation and carbonate alteration at the Shinkai Seep Field, a serpentinite-hosted vent system in the southern Mariana forearc. *Geochem. Geophys. Geosystems* **17**, 3775–3796 (2016).
44. Conti, A., Stefanon, A. & Zuppi, G. M. Gas seeps and rock formation in the northern Adriatic Sea. *Cont. Shelf Res.* **22**, 2333–2344 (2002).

45. Muralidhar, K., Mazumdar, A., Karisiddaiah, S. M., Borole, D. V. & Rao, B. R. Evidences of methane-derived authigenic carbonates from the sediments of the Krishna–Godavari Basin, eastern continental margin of India. *Curr. Sci.* **91**, 6 (2006).
46. Blumenberg, M., Walliser, E.-O., Taviani, M., Seifert, R. & Reitner, J. Authigenic carbonate formation and its impact on the biomarker inventory at hydrocarbon seeps – A case study from the Holocene Black Sea and the Plio-Pleistocene Northern Apennines (Italy). *Mar. Pet. Geol.* **66**, 532–541 (2015).
47. Oppo, D., Capozzi, R., Picotti, V. & Ponza, A. A genetic model of hydrocarbon-derived carbonate chimneys in shelfal fine-grained sediments: The Enza River field, Northern Apennines (Italy). *Mar. Pet. Geol.* **66**, 555–565 (2015).
48. Kase, T., Isaji, S., Aguilar, Y. M. & Kiel, S. A large new Wareniconcha (Bivalvia: Vesicomyidae) from a Pliocene methane seep deposit in Leyte, Philippines. *THE NAUTILUS* **133**, 5 (2019).
49. Kanno, S., Amano, K. & Ban, H. Calyptogena (Calyptogena) pacifica Dall (Bivalvia) from the Neogene system in the Joetsu district, Niigata prefecture. *Trans Proc Palaeont Soc Jpn. New Ser* **153**, 25–35 (1989).
50. Campbell, K. A. Recognition of a Mio-Pliocene Cold Seep Setting from the Northeast Pacific Convergent Margin, Washington, U.S.A. *PALAIOS* **7**, 422 (1992).
51. Nesbitt, E. A. & Campbell, K. A. Spatial and stratigraphic distributions of fossils from diffuse seeps in a Pliocene shelf setting, Cascadia convergent margin. in *Abstracts with Programs* 314 (2004).
52. Cau, S., Franchi, F., Roveri, M. & Taviani, M. The Pliocene-age Stirone River hydrocarbon chemoherm complex (Northern Apennines, Italy). *Mar. Pet. Geol.* **66**, 582–595 (2015).
53. Contardo-Berríos, X., Mena-Hodges, E. & Quiroga-Jamett, E. Finding of the first fossil seep in the emerged coast of Central Chile (33°56' S). Characterization and implications. *Andean Geol.* **44**, 213 (2017).
54. Aiello, I. W., Garrison, R. E., Moore, J. C., Kastner, M. & Stakes, D. S. Anatomy and origin of carbonate structures in a Miocene cold-seep field. 5 (2001).
55. Chien, C.-W., Huang, C.-Y., Chen, Z., Lee, H.-C. & Harris, R. Miocene shallow-marine cold seep carbonate in fold-and-thrust Western Foothills, SW Taiwan. *J. Asian Earth Sci.* **56**, 200–211 (2012).
56. Conti, S., Fontana, D., Lucente, C. C. & Pini, G. A. Relationships between seep-carbonates, mud volcanism and basin geometry in the Late Miocene of the northern Apennines of Italy: the Montardone mélange. *Int. J. Earth Sci.* **103**, 281–295 (2014).
57. Nesbitt, E. A., Martin, R. A. & Campbell, K. A. New records of Oligocene diffuse hydrocarbon seeps, northern Cascadia margin. *Palaeogeogr. Palaeoclimatol. Palaeoecol.* **390**, 116–129 (2013).
58. Miyajima, Y. *et al.* A late Miocene methane-seep deposit bearing methane-trapping silica minerals at Joetsu, central Japan. *Palaeogeogr. Palaeoclimatol. Palaeoecol.* **455**, 1-15 (2016).
59. Aoki, S. Mollusca from the Miocene Kabeya Formation, Joban coal-field, Fukushima Prefecture, Japan. *Sci. Rep. Tokyo Kyoiku Daigaku Sect. C* **17**, 23–40 (1954).
60. Amano, K., Jenkins, R. G., Aikawa, M. & Nobuhara, T. A Miocene chemosynthetic community from the Ogaya Formation in Joetsu: Evidence for depth-related ecologic control among fossil seep communities in the Japan Sea back-arc basin. *Palaeogeogr. Palaeoclimatol. Palaeoecol.* **286**, 164–170 (2010).

61. Saether, K. P. *et al.* New fossil mussels (Bivalvia: Mytilidae) from Miocene hydrocarbon seep deposits, North Island, New Zealand, with general remarks on vent and seep mussels. *Zootaxa* **2577**, 1 (2010).
62. Hirayama, K. Molluscan fauna from the Miocene Hiranita Formation, Chichibu Basin, Saitama Prefecture, Japan. *Tohoku Univ Sci Rep 2nd Ser Geol* **Special Volume n. 6**, 163–177 (1973).
63. Campbell, K. A. Hydrocarbon seep and hydrothermal vent paleoenvironments and paleontology: Past developments and future research directions. *Palaeogeogr. Palaeoclimatol. Palaeoecol.* **232**, 362–407 (2006).
64. Collins, M. A biometric and taxonomic study of Miocene age, hydrocarbon-seep mussels from the East Coast of the North Island, New Zealand. (University of Auckland, 1998).
65. Peckmann, J. *et al.* A Microbial Mat of a Large Sulfur Bacterium Preserved in a Miocene Methane-Seep Limestone. *Geomicrobiol. J.* **21**, 247–255 (2004).
66. Cavagna, S., Clari, P., Dela Pierre, F., Martire, L. & Natalicchio, M. Sluggish and steady focussed flows through fine-grained sediments: The methane-derived cylindrical concretions of the Tertiary Piedmont Basin (NW Italy). *Mar. Pet. Geol.* **66**, 596–605 (2015).
67. Ziegenbalg, S. B. *et al.* Formation of secondary carbonates and native sulphur in sulphate-rich Messinian strata, Sicily. *Sediment. Geol.* **227**, 37–50 (2010).
68. Amano, K. & Jenkins, R. G. New fossil Bathymodiolus (sensu lato) (Bivalvia: Mytilidae) from Oligocene seep-carbonates in eastern Hokkaido, Japan, with remarks on the evolution of the genus. *THE NAUTILUS* **125**, 29–35 (2011).
69. Kiel, S. & Hansen, B. T. Cenozoic Methane-Seep Faunas of the Caribbean Region. *PLOS ONE* **10**, e0140788 (2015).
70. Ricci Lucchi, F. & Vai, G. B. A stratigraphic and tectonofacies framework of the "calcari aLucina" in the Apennine Chain, Italy. *Geo-Mar. Lett.* **14**, 210–218 (1994).
71. Nyman, S. L. & Nelson, C. S. The place of tubular concretions in hydrocarbon cold seep systems: Late Miocene Urenui Formation, Taranaki Basin, New Zealand. *AAPG Bull.* **95**, 1495–1524 (2011).
72. Kanie, Y., Hattori, M. & Sasahara, Y. Two types of white clam communities in Sagami Bay, central Japan: Geologic settings and the Tertiary records in the Miura and Boso Peninsulas. *Sci. Rep. Yokosuka City Mus.* **40**, 37–43 (1992).
73. Gill, F. L., Harding, I. C., Little, C. T. S. & Todd, J. A. Palaeogene and Neogene cold seep communities in Barbados, Trinidad and Venezuela: An overview. *Palaeogeogr. Palaeoclimatol. Palaeoecol.* **227**, 191–209 (2005).
74. Amano, K., Hamuro, T. & Hamuro, M. The Oldest Vesicomyid Bivalves from the Japan Sea Borderland. *Venus* **60**, 198–198 (2001).
75. Conti, S., Fontana, D. & Mecozzi, S. A contribution to the reconstruction of Miocene seepage from authigenic carbonates of the northern Apennines (Italy). *Geo-Mar. Lett.* **30**, 449–460 (2010).
76. Amano, K. & Kiel, S. Two Neogene vesicomyid species (Bivalvia) from Japan and their biogeographic implications. *The Nautilus* **126**, 79–85 (2012).
77. Fontana, D. *et al.* Evidence of climatic control on hydrocarbon seepage in the Miocene of the northern Apennines: The case study of the Vicchio Marls. *Mar. Pet. Geol.* **48**, 90–99 (2013).
78. Kanie, Y. & Sakai, T. Chemosynthetic Thraciid Bivalve NipponothTacia,gen. nov. from the Lower Cretaceous and Middle Miocene Mudstones inJapan. *Venus* **56**, 205–220 (1997).

79. Amano, K. & Ando, H. Giant fossil Acharax (Bivalvia: Solemyidae) from the Miocene of Japan. *THE NAUTILUS* **125**, 207–212 (2011).
80. Amano, K., Jenkins, R. G., Sako, Y., Ohara, M. & Kiel, S. A Paleogene deep-sea methane-seep community from Honshu, Japan. *Palaeogeogr. Palaeoclimatol. Palaeoecol.* **387**, 126–133 (2013).
81. Amano, K., Jenkins, R. G., Ohara, M. & Kiel, S. Miocene vesicomyid species (Bivalvia) from Wakayama in southern Honshu, Japan. *THE NAUTILUS* **128**, 9 (2014).
82. Amano, K. & Kiel, S. Fossil Vesicomyid Bivalves from the North Pacific Region. **49**, 24 (2006).
83. Squires, R. L. & Gring, M. P. Late Eocene chemosynthetic? bivalves from suspect cold seeps, Wagonwheel Mountain, central California. *J. Paleontol.* **70**, 63–73 (1996).
84. Campbell, K. A. & Bottjer, D. J. Fossil cold seeps. *Natl. Geogr. Res. Explor.* **9**, 235–343 (1993).
85. Bojanowski, M. J. Oligocene cold-seep carbonates from the Carpathians and their inferred relation to gas hydrates. *Facies* **53**, 347–360 (2007).
86. Goedert, J. L. & Squires, R. L. First Oligocene Records of Calyptogena (Bivalvia: Vesicomyidae). *Th* Veliger, **36**, 72-77 (1993).
87. Kiel, S. *et al.* Fossiliferous methane-seep deposits from the Cenozoic Talara Basin in northern Peru. *Lethaia* **https://doi.org/10.1111/let.12349** (2019).
88. Hryniewicz, K., Amano, K., Jenkins, R. G. & Kiel, S. Thyasirid bivalves from Cretaceous and Paleogene cold seeps. *ACTA Palaeontol. Pol.* 24 (2017).
89. Harding, I. C. Miocene cold seep faunas and carbonates from Barbados. *Cah. Biol. Mar.*, **39**, 341-344, (1998).
90. Peckmann, J., Goedert, J. L., Heinrichs, T., Hoefs, J. & Reitner, J. The Late Eocene 'Whiskey Creek' methane-seep deposit (western Washington State). *Facies* **48**, 241–253 (2003).
91. Goedert, J. L. & Peckmann, J. Corals from deep-water methane-seep deposits in Paleogene strata of Western Oregon and Washington, U.S.A. in *Cold-Water Corals and Ecosystems* (eds. Freiwald, A. & Roberts, J. M.) 27–40 (Springer, 2005). doi:10.1007/3-540-27673-4_2.
92. Venturini, S., Selmo, E., Tarlao, A. & Tunis, G. Fossiliferous methanogenic limestones in the Eocene flysch of Istria (Croatia). *G. Geol. Ser. 3a* **60**, 219–234 (1998).
93. Tada, T. Geology of Urahoro area, Hokkaido. (Yokohama National University, 2000).
94. De Boever, E., Swennen, R. & Dimitrov, L. Lower Eocene carbonate cemented chimneys (Varna, NE Bulgaria): Formation mechanisms and the (a)biological mediation of chimney growth? *Sediment. Geol.* **185**, 159–173 (2006).
95. Hryniewicz, K. *et al.* Paleocene methane seep and wood-fall marine environments from Spitsbergen, Svalbard. *Palaeogeogr. Palaeoclimatol. Palaeoecol.* **462**, 41–56 (2016).
96. Schwartz, H., Sample, J., Weberling, K. D., Minisini, D. & Moore, J. C. An ancient linked fluid migration system: cold-seep deposits and sandstone intrusions in the Panoche Hills, California, USA. *Geo-Mar. Lett.* **23**, 340–350 (2003).
97. Little, C. T. S. *et al.* Late Cretaceous (Maastrichtian) shallow water hydrocarbon seeps from Snow Hill and Seymour Islands, James Ross Basin, Antarctica. *Palaeogeogr. Palaeoclimatol. Palaeoecol.* **418**, 213–228 (2015).
98. Nobuhara, T. *et al.* Lithofacies and fossil assemblages of the Upper Cretaceous Sada Limenstone, Shimanto City, Kochi Prefecture, Shikoku, Japan. *Foss. Paleontol. Soc. Jpn.* **84**, 47–60 (2008).
99. Shapiro, R. & Fricke, H. Tepee Buttes: Fossilized methane-seep ecosystems. in *GSA Field Guide 3: Science at the Highest Level* vol. 3 94–101 (Geological Society of America, 2002).

100. Kauffman, E. G., Arthur, M. A., Howe, B. & Scholle, P. A. Widespread venting of methane-rich fluids in Late Cretaceous (Campanian) submarine springs (Tepee Buttes), Western Interior seaway, U.S.A. *Geology* **24**, 799–802 (1996).
101. Hepper, K. A new hydrocarbon seep locality in the Mesozoic Great Valley Group, Guenoc Ranch, Northern California. (California State University, San Francisco, 2004).
102. Hikida, Y., Suzuki, S., Togo, Y. & Ijiri, A. An exceptionally well-preserved fossil seep community from the Cretaceous Yezo Group in the Nakagawa area, Hokkaido, northern Japan. *Paleontol. Res.* **7**, 329–342 (2003).
103. Kiel, S. *et al.* Cretaceous methane-seep deposits from New Zealand and their fauna. *Palaeogeogr. Palaeoclimatol. Palaeoecol.* **390**, 17–34 (2013).
104. Liang, H., Chen, X., Wang, C., Zhao, D. & Weissert, H. Methane-derived authigenic carbonates of mid-Cretaceous age in southern Tibet: Types of carbonate concretions, carbon sources, and formation processes. *J. Asian Earth Sci.* **115**, 153–169 (2016).
105. Kanie, Y. & Kuramouchi, T. Description on possibly chemosynthetic bivalves from the Cretaceous deposits of Obira-cho, northwestern Hokkaido. *Sci. Rep. Yokosuka City Mus.* **44**, 63–68 (1996).
106. Campbell, K. A., Farmer, J. D. & Des Marais, D. Ancient hydrocarbon seeps from the Mesozoic convergent margin of California: carbonate geochemistry, fluids and palaeoenvironments. *Geofluids* **2**, 63–94 (2002).
107. Wiese, F., Kiel, S., Pack, A., Walliser, E. O. & Agirrezabala, L. M. The beast burrowed, the fluid followed – Crustacean burrows as methane conduits. *Mar. Pet. Geol.* **66**, 631–640 (2015).
108. Kanie, Y., Yoshikawa, Y., Sakai, T. & Takahashi, T. The Cretaceous chemosynthetic cold water-dependent molluscan community discovered from Mikasa City, central Hokkaido. *Sci. Rep. Yokosuka City Mus.* **41**, 31–36 (1993).
109. Agirrezabala, L. M., Kiel, S., Blumenberg, M., Schäfer, N. & Reitner, J. Outcrop analogues of pockmarks and associated methane-seep carbonates: A case study from the Lower Cretaceous (Albian) of the Basque-Cantabrian Basin, western Pyrenees. *Palaeogeogr. Palaeoclimatol. Palaeoecol.* **390**, 94–115 (2013).
110. Williscroft, K. *et al.* Extensive Early Cretaceous (Albian) methane seepage on Ellef Ringnes Island, Canadian High Arctic. *Geol. Soc. Am. Bull.* **129**, 788–805 (2017).
111. Beauchamp, B., Harrison, J. C., Nassichuk, W. W., Krouse, H. R. & Eliuk, L. S. Cretaceous Cold-Seep Communities and Methane-Derived Carbonates in the Canadian Arctic. *Science* **244**, 53–56 (1989).
112. Reitner, J., Blumenberg, M., Walliser, E.-O., Schäfer, N. & Duda, J.-P. Methane-derived carbonate conduits from the late Aptian of Salinac (Marne Bleues, Vocontian Basin, France): Petrology and biosignatures. *Mar. Pet. Geol.* **66**, 641–652 (2015).
113. Kelly, S. R. A., Blanc, E., Price, S. P. & Whitham, A. G. Early Cretaceous giant bivalves from seep-related limestone mounds, Wollaston Forland, Northeast Greenland. *Geol. Soc. Lond. Spec. Publ.* **177**, 227–246 (2000).
114. Kaim, A., Skupien, P. & Jenkins, R. G. A new Lower Cretaceous hydrocarbon seep locality from the Czech Carpathians and its fauna. *Palaeogeogr. Palaeoclimatol. Palaeoecol.* **390**, 42–51 (2013).
115. Sandy, M. R. *et al.* Methane-seep brachiopod fauna within turbidites of the Sinaia Formation, Eastern Carpathian Mountains, Romania. *Palaeogeogr. Palaeoclimatol. Palaeoecol.* **323–325**, 42–59 (2012).

116. Kiel, S. & Peckmann, J. Paleoecology and Evolutionary Significance Of An Early Cretaceous Peregrinella-Dominated Hydrocarbon-Seep Deposit On The Crimean Peninsula. *PALAIOS* **23**, 751–759 (2008).
117. Kiel, S. *et al.* The Paleoecology, Habitats, and Stratigraphic Range of the Enigmatic Cretaceous Brachiopod Peregrinella. *PLOS ONE* **9**, 19 (2014).
118. Kiel, S. & Campbell, K. A. Lithomphalus enderlini gen. et sp. nov. from cold-seep carbonates in California—a Cretaceous neomphalid gastropod? *Palaeogeogr. Palaeoclimatol. Palaeoecol.* **227**, 232–241 (2005).
119. Hryniewicz, K. *et al.* Late Jurassic–Early Cretaceous hydrocarbon seep boulders from Novaya Zemlya and their faunas. *Palaeogeogr. Palaeoclimatol. Palaeoecol.* **436**, 231–244 (2015).
120. Hryniewicz, K., Hammer, Ø., Nakrem, H. A. & Little, C. T. S. Microfacies of the Volgian-Ryazanian (Jurassic-Cretaceous) hydrocarbon seep carbonates from Sassenfjorden, central Spitsbergen, Svalbard. *Nor. J. Geol.* **92**, 113–131 (2012).
121. Kelly, S. R. A., Ditchfield, P. W., Doubleday, P. A. & Marshall, J. D. An Upper Jurassic methane-seep limestone from the Fossil Bluff Group forearc basin of Alexander Island, Antarctica. *J. Sediment. Res.* **65**, 274–282 (1995).
